# Characteristics of Digital Health Interventions Associated with Improved Glycemic Control in T2DM: A Systematic Review and Meta-Analysis

**DOI:** 10.3390/jcm15031123

**Published:** 2026-01-31

**Authors:** Oscar Eduardo Rodríguez-Montes, María del Carmen Gogeascoechea-Trejo, Clara Bermúdez-Tamayo

**Affiliations:** 1 Universidad Veracruzana, Xalapa 91000, Mexico; orodriguez@adherahealth.com; 2 Universidad de Sevilla, 41004 Sevilla, Spain; 3 Adhera Health SL, 41092 Sevilla, Spain; 4 Institute of Health Sciences, Universidad Veracruzana, Xalapa 91000, Mexico; cgogeascoechea@uv.mx; 5 Department of Applied Economics, University of Granada, 18012 Granada, Spain; 6 Ciber de Epidemiología y Salud Pública—CIBERESP, 08950 Barcelona, Spain; 7 Instituto de Investigación Biosanitaria de Granada (Ibs. Granada), 18012 Granada, Spain

**Keywords:** diabetes mellitus, type 2, telemedicine, mobile applications, remote consultation, self-management, chronic disease, meta-analysis, systematic review, hemoglobin A, glycosylated

## Abstract

**Background/Objective:** Type 2 Diabetes Mellitus (T2DM) represents a major increasing burden for primary care systems worldwide. Digital health interventions (DHIs) have been proposed as scalable tools to improve glycemic control, yet uncertainty remains regarding which intervention characteristics yield the greatest benefit. To evaluate the effectiveness of DHIs on HbA1c levels in adults with T2DM and to examine whether intervention duration, engagement intensity, glucometer integration, and healthcare provider involvement modify glycemic outcomes. Data Sources: PubMed, Embase, Cochrane Library, and JMIR databases were systematically searched for relevant studies published between January 2020 and May 2025. Study Eligibility Criteria: Randomized controlled trials comparing DHIs plus usual care versus usual care alone in adults with T2DM and reporting HbA1c as the primary outcome. **Methods:** Data were extracted using the Jadad scale and TIDieR framework. Random-effects meta-analysis estimated pooled mean differences (MD) in HbA1c with 95% CIs. Subgroup analyses examined effects by intervention characteristics. Heterogeneity and sources of variance were assessed through Cochran’s Q, I^2^, meta-regression, and sensitivity analyses (leave-one-out and trim-and-fill). **Results:** Thirteen RCTs (*n* ≈ 20,000) met inclusion criteria. DHIs achieved significant HbA1c reductions (range 0.01% to 1.57%; pooled MD −1.08%; 95% CI −1.18 to −0.99; *p* = 0.001). Short-term (≤6 months), low-intensity interventions showed the largest effect sizes (MD −1.16%, 95% CI 0.94 to 1.39). Glucometer integration and healthcare provider involvement contributed minimally to additional benefit. Meta-regression confirmed substantial heterogeneity, but no single factor explained variance across studies. Limitations: Considerable heterogeneity across interventions and variability in engagement measurement may limit the generalizability of findings. **Conclusions:** Short-term, low-intensity DHIs significantly improve glycemic control in primary care populations with T2DM. Advanced meta-analytic techniques confirm the robustness of these effects, providing practical guidance for selecting and implementing effective digital interventions in routine diabetes care.

## 1. Introduction

Type 2 Diabetes Mellitus (T2DM) is a prevalent chronic condition affecting more than 537 million adults globally and accounting for more than 90% of all diabetic patients worldwide, with significant economic and healthcare burdens [1]. On average, annual healthcare expenses for individuals with diabetes amount to USD 16,752, more than double that of non-diabetic individuals [2]. Managing T2DM requires long-term interventions, often focusing on reducing glycated hemoglobin (HbA1c) levels, a key indicator of glycemic control.

Given the high costs associated with managing diabetes mellitus, healthcare systems worldwide are increasingly turning to high-impact, low-cost strategies, such as mobile health (mHealth) interventions. Mobile interventions have become increasingly sophisticated in their technology and diverse in their approaches to changing and sustaining health behaviors [3]. Over the past decade, the integration of digital health interventions, particularly mobile health (mHealth) applications, has gained momentum as an innovative approach to improve disease management [4]. DHIs, which are therapies given through digital technology like smartphones or websites, have a huge potential to provide efficient, affordable, safe, and scalable interventions to promote healthcare and can be used to optimize outcomes for chronic diseases [5]. Health systems worldwide are increasingly seeking digital health strategies that are both effective and cost-efficient, particularly in developing countries where dedicated digital health departments are often lacking. The key for different governments worldwide to support the use of digital health or mobile health is based on a cost-effectiveness relationship, to be able to implement public policies that allow the design, creation, use, evaluation, and maintenance of software or apps.

The World Health Organization [6] has highlighted digital health as a key component of health system transformation, emphasizing that implementation should be evidence-based and supported by adequate workforce skills and professional competencies [4]. Newer digital health tools increasingly incorporate data-driven personalization features to enhance patient engagement and adherence [7,8].

Despite the increasing use of digital interventions, their effectiveness in reducing HbA1c levels in patients with T2DM remains uncertain. Previous systematic reviews have examined digital health interventions, but many have lacked specificity in focusing on randomized clinical trials (RCTs) and use glycemic control as a primary outcome [9,10,11,12,13]. Moreover, the existing literature presents significant variability in the design, duration, and adherence to digital interventions, making it difficult to draw conclusive evidence regarding their efficacy in T2DM management. This systematic review addresses a specific knowledge gap by providing evidence on the impact of interventions on HbA1c levels in T2DM patients.

## 2. Materials and Methods

This systematic review follows the PRISMA 2020 guidelines [14], and the completed PRISMA checklist is provided in the Supplementary Materials S1; the protocol was registered on the Open Science Framework (OSF): https://osf.io/kbnwq/, accessed on 7 July 2025. The study aims to evaluate the effectiveness of digital health interventions in reducing HbA1c levels among patients with T2DM, focusing on the characteristics associated with improved outcomes. Specifically, it will (1) assess the impact of intervention duration, comparing short-term (≤6 months) and long-term (>6 months) interventions; (2) examine the role of glucometer integration within the digital intervention (telemonitoring, mobile apps, SMS, and web-based platforms) and its correlation with HbA1c reduction; (3) investigate whether interventions involving healthcare provider support (reported as professionals from any area of health who participated in the design and monitoring of the intervention) differ in effectiveness from those without a reported provider involvement; and (4) analyze the impact of intervention intensity, categorized as low, moderate, or high, on HbA1c reduction, to identify the optimal engagement level for glycemic control.

### 2.1. Eligibility Criteria

We included studies that met the following criteria: (1) Study design: Randomized controlled trials (RCTs). (2) Participants: Patients included were diagnosed with T2DM. (3) Intervention: Digital interventions, including mobile health (mHealth) applications, aimed at influencing HbA1c levels. (4) Outcome: The primary outcome must be the measurement of HbA1c levels. (5) Comparator: (standard treatment + use of the app) and control group (standard treatment) were compared. (6) Publication Date: Studies published between January 2020 and May 2025 were considered for inclusion, reflecting the period of rapid evolution in mHealth technologies. The rationale for this cutoff is that technological advances in AI and digital health interventions have significantly evolved since 2020, making earlier studies less relevant to current healthcare practices. The post-2020 period also reflects a substantial increase in the development and adoption of mHealth technologies [15]. Language: Studies included were published in English or Spanish.

### 2.2. Information Sources

A systematic search was conducted in the following electronic databases: PubMed, Embase, Cochrane, and JMIR search portals as bibliographic databases.

### 2.3. Search Strategy

The search strategy followed the PICO-SD framework: Population (P): Adults with Type 2 Diabetes Mellitus; Intervention (I): Digital health interventions (telemonitoring, mobile apps, SMS, and web-based platforms); Comparator (C): Usual or non-digital care; Outcomes (O): Primary outcome—HbA1c reduction, and secondary—other glycemic indicators; Study Design (SD): Randomized controlled trials, including cluster-RCTs.

A comprehensive search strategy was developed combining free-text terms and Medical Subject Headings (MeSHs), structured around four main concepts: (1) condition of interest (Type 2 Diabetes), (2) digital interventions, (3) study design, and (4) primary outcome. The following terms were used: Condition (Type 2 Diabetes): “type 2 diabetes”, “diabetes mellitus”, “diabetes mellitus type II”, “diabetes type 2”, “type II diabetes mellitus”, “DM2”, “non insulin dependent”, “non-insulin dependent”, “noninsulin dependent diabetes”, “adult onset diabetes”, and “maturity onset diabetes”. Digital Interventions: “digital app”, “digital application”, “mobile health”, “mHealth”, “m-health”, and “diabetes apps”. Study Design: “clinical trial”, “randomized controlled trial”, “RCT”, “efficacy”, and “effectiveness”. Primary Outcome (Glycemic Control): “HbA1c”, “hemoglobin A1c”, “glycosylated hemoglobin”, “glycated hemoglobin”, and “HbA1c value”.

The detailed search strategy, including Boolean operators and database-specific adaptations, is provided in the Supplementary Materials S2.

### 2.4. Study Selection

Two independent reviewers screened the titles and abstracts of identified studies. Full-text articles were assessed for eligibility based on predefined criteria. Disagreements were resolved through discussion or consultation with a third reviewer. A PRISMA flow diagram was used to illustrate the study selection process.

### 2.5. Data Collection Process

Data from the included studies were extracted using a standardized data extraction form in Excel. The extracted data included study characteristics (author, year, and country), population demographics, details of the intervention and control groups, study duration, outcome measures (HbA1c levels), and key findings.

Interventions were classified as low intensity (≤1 contact per month or total contact time <3 h), moderate intensity (2–3 contacts per month or 3–10 h in total), or high intensity (≥1 contact per week or >10 h in total) during the intervention period.

To ensure comprehensive reporting of the intervention, we utilized the Template for Intervention Description and Replication (TIDieR) checklist. This tool provides a structured framework for detailing interventions, enhancing transparency, and facilitating replication in future studies. By adhering to TIDieR guidelines, we systematically described the intervention’s components, including its rationale, materials, procedures, delivery methods, and any modifications made during the study. The completed TIDieR checklist is provided in the Supplementary Materials S3.

### 2.6. Risk of Bias Assessment

The risk of bias for the included RCTs was assessed using the Jadad scale [15], which evaluates three key methodological domains: adequacy of randomization, blinding, and reporting of withdrawals or dropouts. In its modified form, the scale also incorporates stricter criteria by allowing the subtraction of one point if randomization or blinding procedures are judged inadequate, while the total score remains within the 0–5 range. The complete Jadad scale and scoring criteria used in this review are provided in the Supplementary Materials S4.

Trials with a score of ≥3 were considered to have a low risk of bias, whereas those scoring lower were classified as high risk. This threshold is widely accepted as an indicator of methodological soundness. Applying this criterion ensured that the evidence synthesis was based on studies with higher internal validity, thereby enhancing the robustness and credibility of the review findings.

The Jadad scale offers a practical and straightforward approach that facilitates consistent, transparent, and reproducible assessment of study quality in systematic reviews and meta-analyses. Its use in this review contributed to strengthening the methodological rigor and reliability of the conclusions drawn.

### 2.7. Data Synthesis

A narrative synthesis was first conducted to summarize study characteristics, intervention details, and outcomes not suitable for pooling. This was complemented by a meta-analysis estimating pooled mean differences in HbA1c with 95% Confidence Intervals across eligible trials.

### 2.8. Data Analysis

The key outcome of interest was the change in HbA1c levels, expressed as mean differences (MD) between intervention and control groups. A random-effects inverse-variance model with DerSimonian–Laird (DL) estimate of tau^2^ was used to pool the effect sizes. To assess the robustness of the pooled estimates, sensitivity analyses were conducted using an alternative estimator of between-study variance (restricted maximum likelihood, REML). The direction and magnitude of the pooled effect sizes for HbA1c reduction remained consistent with the primary DerSimonian–Laird model, and no relevant changes were observed in heterogeneity estimates or statistical significance.

Subgroup analysis was performed based on the duration of the interventions, categorizing them into 2 groups: short-term (six months or less) and long-term (more than six months). Other subgroups include the following: use of a glucometer (with or without), the presence of health care providers for the design and follow-up of the interventions (described or not described), and intensity (low, medium, and high) according to the design of the interventions. We reported effect estimates with 95% Confidence Intervals (CIs), and heterogeneity was assessed using Cochran’s Q statistic and I^2^ values.

Meta-regression analysis was performed to explore potential sources of heterogeneity across the included studies. The goal of the meta-regression was to examine the relationship between the effect sizes (HbA1c reduction) and specific study-level covariates, which could help explain the variation in the effectiveness of digital educational interventions for diabetes management. (1) Standard Error (SE): This accounted for the precision of each study’s effect estimate; (2) Initial HbA1c: Baseline levels of glycated hemoglobin reflected the glycemic control at the start of the study; (3) Total Sample Size: The number of participants in each study was used to assess whether the effect size varied with sample size; (4) Year of Publication (year): The year in which the study was published is relevant for examining whether there was any temporal trend in the reported effect sizes.

We used a random-effects meta-regression model, which incorporates the variability between studies and accounts for both within-study and between-study heterogeneity. The random-effects model was chosen due to the significant heterogeneity observed in the effect sizes across the included studies, as indicated by the high I^2^ in the initial meta-analysis. The meta-regression was performed using the restricted maximum likelihood (REML) method to estimate between-study variance (tau^2^). This method provides an unbiased estimate of the variance components in the presence of small samples and accounts for both observed covariates and unexplained heterogeneity.

The heterogeneity was assessed using the following metrics: Tau^2^ (between-study variance) is an estimate of the between-study variability; I^2^ is the percentage of residual variation due to heterogeneity, indicating the proportion of total variability in effect estimates that is due to heterogeneity rather than chance.

A leave-one-out sensitivity analysis was performed to assess the robustness of the pooled effect size from the meta-analysis. This analysis involved systematically omitting each study from the meta-analysis and recalculating the pooled effect estimate to evaluate the influence of individual studies on the overall result.

Finally, given the high heterogeneity observed in the meta-analysis, there was a concern that potential publication bias could be contributing to the variability in effect sizes. To address this, a trim-and-fill analysis was conducted. This method estimates the number of potentially missing studies due to publication bias and adjusts the meta-analysis results accordingly. It is widely applied in meta-analyses. This approach detects potential asymmetry in funnel plots and estimates the impact of possibly missing studies by providing an adjusted pooled effect size. The initial analysis was performed using a random-effects model. The trim-and-fill analysis applied a linear trimming estimator to identify any missing studies.

## 3. Results

### 3.1. Study Selection

At the end of the search, a total of 185 articles were reviewed, of which 18 duplicates were eliminated for a total of 167.

From this first partial cut, 134 out of 167 studies were eliminated for having assessments of other objectives, a study design different from a randomized clinical trial, systematic reviews, traditional or non-digital health interventions, evaluation of another type of diabetes, pilots, or research protocols were rejected.

**Figure 1:**
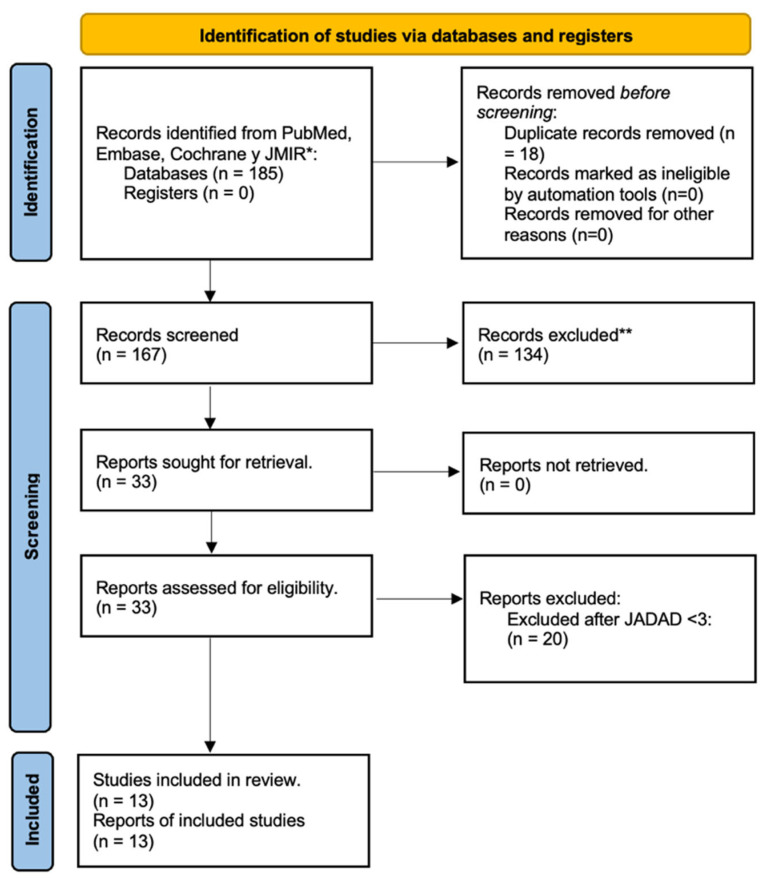
Diagram PRISMA of identification, screening, eligibility, and inclusion of original articles. * Pubmed (*n* = 96); Embase (*n* = 48); Cochrane (*n* = 24); and JMIR (*n* = 17). ** Reasons for exclusion: Study design (*n* = 29); scoping review (*n* = 24); non-digital interventions (*n* = 17); T1DM evaluated (*n* = 15); protocols (*n* = 10); prediabetes (*n* = 3); and gestational diabetes (*n* = 3).

Subsequently, these 33 articles were subjected to a specific review according to the main objectives of this study, (Figure 1), and all of them met the following inclusion criteria: (1) study design and randomized clinical trials; (2) digital health diabetes intervention with the use of apps or software based on mHealth; (3) evaluation of effectiveness on Hb1Ac levels; (4) articles published in 2020 to may 2025; and (5) original articles.

For the final part of the analysis, the Jadad scale was applied to assess the risk of bias [16] for the included RCTs; this approach ensured the inclusion of only 13 high-quality studies.

#### Description of Studies and Patients’ Characteristics

The 13 included studies were conducted across various countries, including the USA [4], China [5], Spain [1], South Korea [1], Japan [1], and Australia [1], with sample sizes ranging from 50 to over 17,000 participants. The 13 selected articles and their main characteristics are presented in the following table (Table A1 in the Appendix A).

### 3.2. Intervention Characteristics

A total of 13 interventions were analyzed using the Tidier scale, Table A2 (see Appendix A). Among these, six interventions (46.15%) utilized a glucometer for monitoring and self-management of blood glucose levels [16,17,18,19,20,21]. These interventions typically involved cellular-enabled glucose meters or similar technologies integrated into mobile health systems. In contrast, seven digital health interventions (53.85%) did not include a glucometer, focusing instead on educational programs, app-based coaching, or telemedicine without direct blood glucose monitoring [22,23,24,25,26,27,28].

The interventions were delivered by a variety of providers. In six cases (46.2%), interventions were provided by healthcare professionals such as physicians, nurse educators, dietitians, or psychologists [17,19,20,26,27,28]. In the remaining seven interventions (53.9%), the delivery was automated through mobile apps or telemedicine platforms without direct involvement of healthcare providers [16,18,21,22,23,24,25].

The intensity of the interventions varied. Three interventions (23.1%) were highly intensive, involving daily or bi-weekly monitoring and feedback [16,20,21]. Another five interventions (38.5%) had a moderate intensity, with participants receiving weekly or monthly guidance and monitoring [17,22,26,27,28]. The remaining five interventions (38.5%) were lower in intensity, with participants interacting with the intervention only once or at predefined intervals [18,19,23,24,25].

### 3.3. Risk of Bias

The overall results indicate a variation in the methodological quality of the studies, as reflected in their Jadad scores (see Supplementary Materials). The majority of studies scored 3 or higher, suggesting an overall moderate to high quality in the assessment of randomization, blinding, and dropout reporting across the studies included in this review. However, certain aspects, particularly blinding and the adequacy of randomization, require attention in future studies to ensure more robust methodological designs.

Two studies, Livongo [17] and My Diabetes Coach [23], had lower quality based on incomplete descriptions of randomization and inadequate or absent blinding. The Livongo study had no blinding or adequate randomization, scoring negatively. Several other studies, such as TangPlan and WeChat [18], Kaisang’s intervention [25], and Indica STUDY [27], also scored 3, mostly due to the lack of blinding and limited reporting on withdrawals and dropouts. Other studies [22,26,29] had Jadad scores of 4 and 5, indicating an improvement in methodology, especially with the appropriate reporting of randomization and some level of blinding, but still lacking in one or more criteria such as blinding adequacy or dropout reporting.

The studies IMB Model [24], EPICC [28], SINOMEDISITE [19], and ROADMAP [21] scored 6, demonstrating high methodological rigor. These studies had clear descriptions of randomization methods, adequate blinding procedures, and proper reporting of withdrawals or dropouts, making them the most methodologically sound studies in this review. The certainty of evidence for the primary outcome (HbA1c reduction) was assessed using the GRADE approach. The complete GRADE Summary of Findings table is provided in the Supplementary Materials S5.

### 3.4. Meta-Analysis

Primary Analyses

A total of 13 studies were included in the meta-analysis. The overall effect estimate for the change in HbA1c across the studies was −1.08% (95% CI: −1.18 to −0.96), suggesting a positive and significant reduction in HbA1c levels (*p* = 0.001).

### 3.5. Study-Level Results

**Figure 2:**
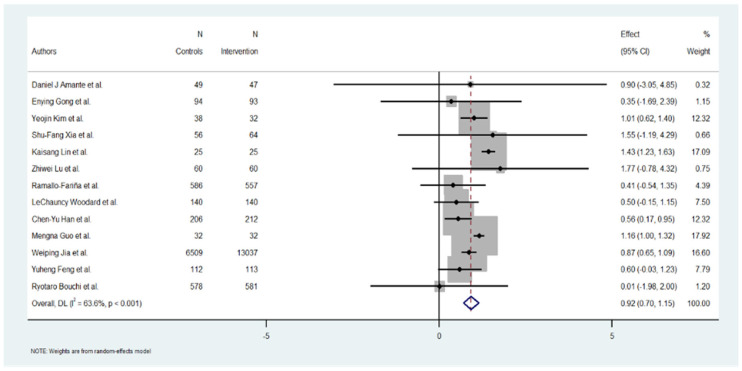
Effect of Hb1Ac reduction by digital intervention [17,18,19,20,21,22,23,24,25,26,27,28,29].

The individual effect estimates varied widely across the included studies. Yeojin Kim et al. [24] reported a significant HbA1c reduction of 1.01 (95% CI: 0.62 to 1.40), contributing 12.32% to the overall weight. Kaisang Lin et al. [25] also reported a significant reduction of 1.43 (95% CI: 1.23 to 1.63), contributing 17.09% to the weight. Mengna Guo et al. [20] showed an effect of 1.16 (95% CI: 1.00 to 1.32), with 17.92% of the weight. Conversely, Ryotaro Bouchi et al. [22] demonstrated a minimum effect of 0.01 (95% CI: −1.98 to 2.00), contributing 1.20% to the total analysis. Several studies, such as those by Daniel J Amante et al. [17], Enying Gong et al. [23], and Zhiwei Lu [26] et al., reported moderate to good results in HbA1c reduction but a low level in the % of weight. Overall, the heterogeneity of the effect sizes was high, as reflected in the heterogeneity measures below (Figure 2).

### 3.6. Heterogeneity Analysis

The heterogeneity among the included studies was substantial, as evidenced by the following measures: Cochran’s Q = 33.01, with a *p*-value < 0.001, indicating significant heterogeneity. I^2^ = 84.68%, suggesting that almost all the observed variability in the effect sizes can be attributed to between-study differences rather than chance.

The heterogeneity variance (tau^2^) was estimated at 0.0674. The high levels of heterogeneity (I^2^ > 84.8%) indicate considerable variability across the studies in the reported effects of digital educational interventions on HbA1c reduction. Categorical variables such as provider type and intervention modality were incorporated into subgroup analyses, which help to explore but cannot fully account for the observed heterogeneity. This should be considered when interpreting the variability of effects across trials.

Specifically, the digital interventions evaluated demonstrated highly variable effects on HbA1c reduction, reflecting differences in intervention design and implementation. In addition, the studies encompassed a wide range of sample sizes, from small pilot trials to large-scale randomized controlled trials, which may have disproportionately influenced the pooled estimates. Furthermore, substantial variability was present in the duration of interventions and length of follow-up, thereby increasing between-study variability and contributing to the overall heterogeneity detected.

The high heterogeneity (I^2^ > 80%) likely reflects differences in population characteristics, intervention design, and follow-up duration, which could not be fully explained by subgroup or meta-regression analyses. These factors should be considered when interpreting the pooled estimates.

### 3.7. Subgroup Analysis

**Figure 3:**
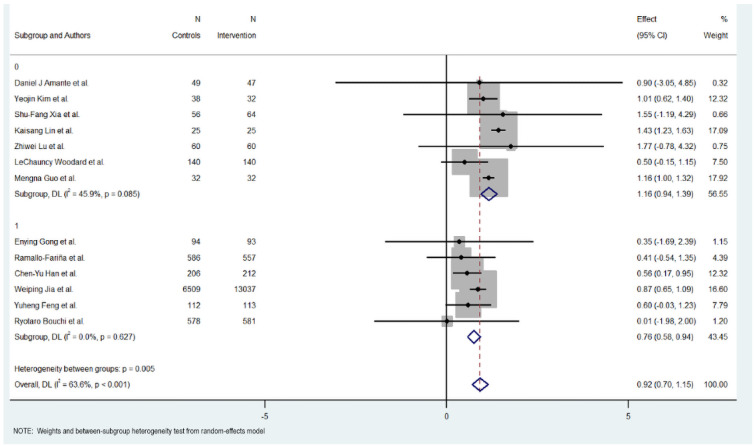
HbA1c reduction according to duration in digital health interventions [17,18,19,20,21,22,23,24,25,26,27,28,29].

**Figure 4:**
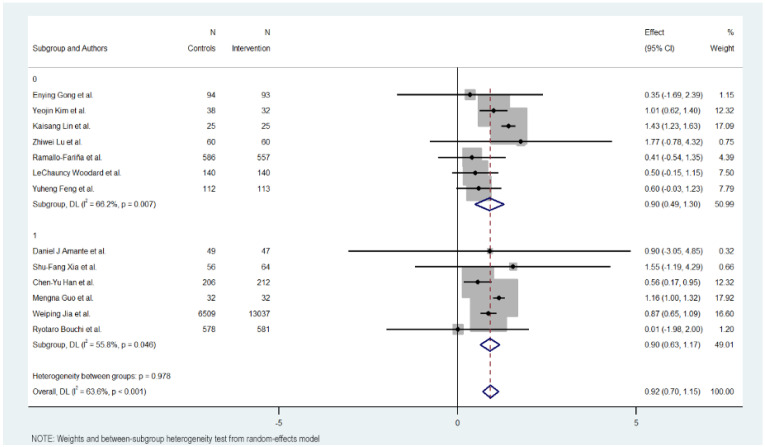
HbA1c reduction according to intensity in digital health interventions [17,18,19,20,21,22,23,24,25,26,27,28,29].

**Figure 5:**
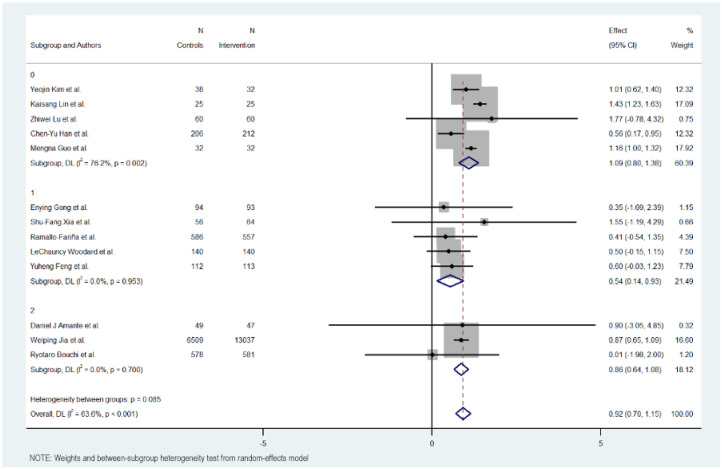
HbA1c reduction according to glucometer integration in digital health interventions [17,18,19,20,21,22,23,24,25,26,27,28,29].

**Figure 6:**
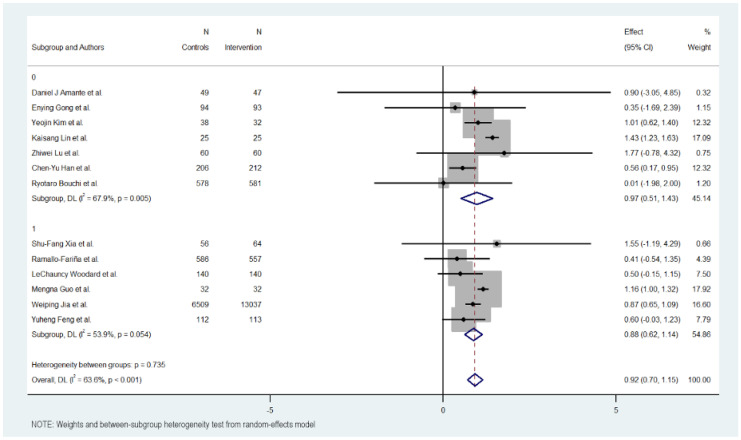
HbA1c Reduction according to healthcare provider involvement in digital health interventions [17,18,19,20,21,22,23,24,25,26,27,28,29].

Figure 3, Figure 4, Figure 5 and Figure 6 illustrate the impact of key design features of digital health interventions on HbA1c reduction: differences according to intervention duration (short-term vs. long-term) and intensity (low, moderate, and high), the effects of glucometer integration, and the involvement of healthcare providers.

### 3.8. Duration of Intervention

Subgroup analysis revealed differences between short-term (six months or less) and long-term interventions (more than six months). For short-term interventions, the pooled effect size was significant, with an MD of 1.165 (95% CI: 0.94 to 1.390, *p* < 0.000), indicating a positive impact of the digital interventions on reducing HbA1c levels. The short-term interventions exhibited regular heterogeneity (Cochran’s Q = 11.10, *p* = 0.085, I^2^ = 45.9%).

Meanwhile, long-term interventions did not show a statistically significant effect on HbA1c reduction (MD = 0.758, 95% CI: 0.581 to 0.935, *p* = 0.000). Heterogeneity in this subgroup was low (Cochran’s Q = 3.48, *p* < 0.627, I^2^ = 0.0%), suggesting no substantial variability among studies in this group.

Overall, the heterogeneity was moderate and significant with all included studies (Cochran’s Q = 33.01, *p* < 0.001, I^2^ = 63.6%), indicating variations in the effects of digital educational interventions on HbA1c reduction. However, significant heterogeneity was also detected between the short-term and long-term intervention subgroups (Cochran’s Q = 7.74, *p* < 0.005), with short-term interventions showing a more consistent and favorable outcome.

### 3.9. Intensity of Intervention

The intensity of the groups was also studied in three groups: low, moderate, and high intensity in sending content and messages.

The subgroup with a low intensity of the intervention obtained better results in reducing Hb1Ac, followed by the subgroup with a high intensity. The subgroup with a moderate intensity was the one that showed the least effect in reducing Hb1Ac.

The subgroup with a low intervention intensity had an MD of 1.091 (95% CI: 0.801 to 1.381, *p* < 0.000), indicating more than 1% on Hb1Ac levels. This subgroup exhibited high heterogeneity (Cochran’s Q = 16.83, *p* = 0.002, I^2^ = 76.2%).

The subgroup with a moderate intervention intensity had an MD of −0.540 (95% CI: −0.934 to −0.145; *p* < 0.007), indicating a modest reduction in Hb1Ac, half the effect compared to the low intensity intervention. The heterogeneity in this group was low (Cochran’s Q = 0.68, *p* = 0.953, I^2^ = 0.0%).

The subgroup with an intense intervention intensity had an MD of 0.860 (95% CI: 0.644 to 1.076, *p* < 0.000), indicating a midpoint effect between low and moderate intensity. The heterogeneity in this group was also low (Cochran’s Q = 0.71, *p* = 0.700, I^2^ = 0.0%).

Overall, the heterogeneity was moderate and significant with all included studies (Cochran’s Q = 33.01, *p* < 0.001, I^2^ = 63.6%), indicating variations in the intensity of digital educational interventions on HbA1c reduction. In addition, significant heterogeneity was not detected between the level of intensity intervention subgroups (Cochran’s Q = 4.93, *p* < 0.085), with interventions with lower intensity showing a more favorable outcome compared to the others.

### 3.10. Use of Glucometer

The use of a glucometer revealed no differences between interventions with or without it. The interventions without using a glucometer had 0.895 effect against 0.902 in the interventions using it.

The interventions without a glucometer had an MD of 0.895 (95% CI: 0.488 to 1.302, *p* < 0.000), indicating a positive impact of the digital interventions on reducing HbA1c levels. These interventions exhibited moderate heterogeneity (Cochran’s Q = 17.73, *p* = 0.007, I^2^ = 66.2%).

Meanwhile, interventions with a glucometer show almost the same effect on HbA1c reduction (MD = 0.902, 95% CI: 0.629 to 1.175, *p* = 0.000). Heterogeneity in this subgroup was low (Cochran’s Q = 11.30, *p* < 0.046, I^2^ = 55.8%).

Overall, the heterogeneity was moderate and significant with all included studies (Cochran’s Q = 33.01, *p* < 0.001, I^2^ = 63.6%), indicating variations in the effects of digital educational interventions on HbA1c reduction. In addition, significant heterogeneity was not detected between the short-term and long-term intervention subgroups (Cochran’s Q = 0.00, *p* < 0.978), with interventions with a glucometer showing a more favorable outcome but not showing a big difference.

### 3.11. Providers

Analysis revealed little differences between interventions in which it is indicated were designed and supervised by health professionals compared to those that do not specify it.

The group of interventions where the participation of health providers is described showed a lower effect with an MD of 0.877 (95% CI: 0.618 to 1.136, *p* < 0.000). These interventions exhibited moderate heterogeneity (Cochran’s Q = 10.85, *p* = 0.054, I^2^ = 53.9%).

On the other hand, the group of interventions that do not describe the participation of health providers showed a better effect with an MD of 0.968 (95% CI: 0.509 to 1.427, *p* < 0.000). These interventions exhibited moderate heterogeneity (Cochran’s Q = 18.69, *p* = 0.005, I^2^ = 67.9%).

Overall, the heterogeneity was moderate and significant with all included studies (Cochran’s Q = 33.01, *p* < 0.001, I^2^ = 63.6%), indicating variations in the effects of provider support interventions on HbA1c reduction. However, significant heterogeneity was not detected between the provider and non-provider intervention subgroups (Cochran’s Q = 0.11, *p* 0.735), with better results in the subgroup that does not describe the use of health providers for the design and supervision of the intervention.

### 3.12. Metaregresion

The meta-regression revealed that none of the individual covariates, including initial HbA1c, sample size, or year of publication, significantly explained the variation in effect sizes across the studies. Although the model accounted for about 29% of the between-study variance, a large proportion of the heterogeneity (96.81%) remains unexplained, indicating that other factors may contribute to the variability in the effectiveness of digital interventions for glycemic control in diabetes management.

The coefficient for initial HbA1c was −0.333 (95% CI: −0.9144903 to 0.248321, *p* = 0.211). This suggests a negative but non-significant relationship between initial HbA1c levels and the effect size. The coefficient for total sample size was 0.1866961 (95% CI: −1.17228 to 1.545672, *p* = 0.748). This indicates that the total number of participants in the studies had no significant effect on the effect size. The coefficient for the year of publication was 0.1093 (95% CI: −0.2555 to 0.4742, *p* = 0.491). This suggests a positive relationship between the year of publication and the effect size, although this was not statistically significant.

### 3.13. Leave-One-Out Sensitivity Analysis

The overall pooled effect estimate from the meta-analysis was 1.084 (95% Confidence Interval [CI]: 0.988 to 1.180). The sensitivity analysis revealed that the pooled effect size remained relatively stable when individual studies were excluded, suggesting that no single study had a disproportionately large influence on the overall estimate.

### 3.14. Cumulative Meta-Analysis by Year

The cumulative analysis showed variable levels of heterogeneity across the years. Initially, heterogeneity was minimal, with I^2^ = 0.0% for the first two studies. However, as more studies were included, particularly from 2021 onwards, the heterogeneity increased moderately before dropping again to negligible levels by 2024.

### 3.15. Meta-Analysis and Trim-And-Fill Analysis

A trim-and-fill analysis was conducted to assess and adjust for publication bias. The linear trimming estimator identified no missing studies; the between-study variance (tau^2^) is 0.067, indicating no substantial reduction in heterogeneity after adjusting for potential publication bias.

## 4. Discussion

### 4.1. Principal Findings and Comparison with Previous Studies

This systematic review aimed to evaluate the effectiveness of digital interventions, specifically mobile health (mHealth) applications, in reducing HbA1c levels in patients with T2DM. The results demonstrated that mHealth interventions have the potential to significantly lower HbA1c levels, with reductions ranging from 0.35% to 1.57%. However, the findings also highlight the substantial variability in the effectiveness of these interventions, depending on factors such as intervention duration, user adherence, and the specific design of the digital tools.

The results of this review are consistent with previous studies that have reported moderate improvements in glycemic control through digital interventions [9,10,11,12]. However, our analysis further emphasizes the critical importance of the duration of the intervention and user engagement. Interventions that lasted six months or less consistently achieved greater reductions in HbA1c levels compared to longer interventions. These findings suggest that sustained engagement with mHealth tools may be essential for achieving optimal outcomes in glycemic control. Other systematic reviews have found a decrease in Hb1Ac levels with better results in interventions of average duration between 12 and 24 weeks [30]. Meanwhile, interventions targeting HbA1c reduction with the support of telemedicine during the COVID-19 pandemic demonstrated a pooled mean decrease of −0.6% relative to usual care [13].

Subgroup analysis revealed that interventions ≤ 6 months with lower intensity achieved the most substantial reductions in HbA1c levels, suggesting that brief, concentrated engagements may optimize glycemic outcomes in Type 2 Diabetes management. These findings align with previous studies indicating that targeted, time-bound interventions can enhance patient adherence and engagement, leading to measurable improvements in glycemic control [17,19]. The second group with higher intensity achieved the second most substantial reductions, and the third group with moderate intensity the lowest, although other studies show better results in interventions with moderate intensity, the results in high- and low-density interventions obtain very similar results [11]. Conversely, the inclusion of glucometer use and direct involvement of healthcare providers showed minimal impact on HbA1c reduction, highlighting that intervention effectiveness may hinge more on the frequency and intensity of patient engagement than on the addition of specific monitoring tools or professional oversight [9,29]. The results change when the digital intervention includes, in addition to the use of the application, the participation of health coaches who carry out continuous monitoring and intervention in patients, where results can be seen not only in the decrease in glycated hemoglobin, but also in other clinical indicators such as high blood pressure and BMI, as well as in behavioral changes in food choices [10]. It is also important to note that the limited additional benefit observed from healthcare provider involvement and glucometer integration may depend on contextual factors such as the intensity of patient engagement and the design of feedback mechanisms. These components may enhance adherence or motivation in some settings but not necessarily translate into greater glycemic improvements across all interventions. During the pandemic, some studies with subgroup analyses indicated greater efficacy in trials implementing frequent and personalized monitoring, as well as in those delivering high-intensity interventions. All this suggests that a scalable, self-directed intervention model focusing on high user engagement and structured, short-term content delivery may serve as an efficient approach for glycemic management in broader healthcare settings [11].

The heterogeneity in the design of the interventions, including differences in the functionalities of the mHealth apps, participation of clinicians, or additional communication tools such as those provided by health coaches, also played a role in the variability of results. While some apps focused on self-management support through reminders and educational content, others incorporated more advanced features such as real-time feedback, artificial intelligence, or telemedicine consultations. As we discussed, the latter group of interventions tended to report better outcomes, reinforcing the value of integrating more personalized and adaptive digital features into diabetes management tools.

### 4.2. Strengths and Limitations

The findings of this review suggest that mHealth interventions, particularly those incorporating advanced features such as real-time feedback and AI-driven recommendations, can be effective tools for improving glycemic control in patients with Type 2 Diabetes Mellitus (T2DM). Healthcare providers should consider integrating these digital tools into routine diabetes care, especially for patients who require additional support in self-management.

By focusing exclusively on studies with rigorous experimental designs, this review aims to provide a clearer understanding of the effectiveness of digital health technologies in improving glycemic control. This information is essential at a time when the adoption of digital tools in healthcare is growing rapidly. The findings will add to the existing academic literature on digital health interventions and provide practical evidence-based information for healthcare providers and policy makers seeking to implement these technologies to improve diabetes management and control. DHIs encompass all types of interventions with characteristics and different ways in which digital and mobile technologies are being used to support health system needs. Unlike other systematic reviews and meta-analyses, our research is based on studying the most relevant characteristics of digital health interventions in users with T2DM based on randomized clinical trials with a control and experimental group, considering Hb1Ac as the main indicator of efficacy but assessing strategic points of the interventions such as duration, intensity, use of a glucometer, and the participation of health professionals in the design or monitoring of the digital intervention. Features that will allow for the selection of digital tools with significant results, but also enable public decision-makers to select those they consider optimal based on the context and objectives they pursue in public health.

However, the variability in outcomes underscores the importance of personalized approaches, tailoring digital interventions to individual needs and preferences. From a practical perspective, these findings indicate that low- to moderate-intensity interventions of adequate duration may be feasibly implemented in routine diabetes care, particularly in primary care settings with limited resources. Clearly specifying session frequency, follow-up timing, and comparator conditions can help guide providers in selecting and adapting digital health strategies to their patient populations.

Policymakers should also be aware of the potential of digital health technologies to reduce healthcare costs by improving disease management. However, given the variability in outcomes, it is crucial to establish standardized guidelines for the development, implementation, and evaluation of these tools to ensure their effectiveness across diverse populations.

Despite the promising results, this review has several limitations. First, the studies included varied considerably in terms of sample size, intervention duration, and study settings, which may affect the generalizability of the findings. Additionally, most studies were conducted in high-income countries, which may limit the applicability of the results to low- and middle-income settings where access to digital tools and healthcare infrastructure differs. Another limitation is the lack of long-term follow-up data in most studies. While short-term improvements in HbA1c are valuable, diabetes management is a chronic, lifelong process, and it remains unclear whether the benefits observed in these studies can be sustained over the long term. The quality of the selected articles also turned out to be a limitation. Although 33 articles were selected after the bibliographic search and the elimination of exclusion criteria according to the objectives of the study, only 13 were selected after being reviewed with the modified Jadad scale. Finally, we found a limitation in the study of subgroups of health providers, because no description was found in a group of interventions of the participants in the design and follow-up of their RCTs, probably assuming that the design of the interventions and follow-up were carried out by health professionals.

Future research should prioritize long-term evaluations to better assess the durability and sustainability of the effects of digital health interventions in diabetes management. Although no substantial publication bias was detected in the present analysis, the possibility that studies reporting positive outcomes are more likely to be published than those with null or negative results cannot be fully excluded. In addition, while the use of the modified Jadad scale ensured the inclusion of randomized controlled trials with acceptable methodological quality, the inherent subjectivity of risk-of-bias assessment tools may have introduced some variability in study selection.

Further studies should also explore the incremental value of emerging digital features, including machine learning–driven personalization and telemedicine-based support, to determine their added clinical benefit beyond conventional digital interventions. Particular emphasis should be placed on strategies to sustain long-term user engagement, which remains a critical determinant of glycemic outcomes and overall intervention effectiveness.

## 5. Conclusions

This systematic review and meta-analysis confirm that digital health interventions (DHIs), particularly short-term and low-intensity mHealth applications, significantly reduce HbA1c levels in patients with Type 2 Diabetes. The finding that simple, low-intensity interventions can achieve meaningful glycemic improvements is especially relevant in resource-limited settings, where scalability and cost are critical. The use of glucometers and healthcare provider involvement showed minimal additional benefit, suggesting that effective diabetes management can be achieved through self-directed, digitally supported care models. These findings indicate that intervention duration and intensity are the main factors associated with improved glycemic outcomes.

Given the heterogeneity among studies and the limited long-term follow-up, the results should be interpreted with caution. Future research is needed to confirm the sustainability and applicability of these low-resource digital strategies through long-term, high-quality trials.

## Data Availability

No new data were created or analyzed in this study.

## Supplementary Materials

The following supporting information can be downloaded at: https://www.mdpi.com/article/10.3390/jcm15031123/s1, Supplementary Materials S1: PRISMA Checklist; Supplementary Materials S2: Searching strategy; Supplementary Materials S3: Template for the checklist for the description and replication of interventions (tidier); Supplementary Materials S4: Jadad scores for selected articles; Supplementary Materials S5: Grade summary.

## Abbreviations

The following abbreviations are used in this manuscript:

AI	Artificial Intelligence
CI	Confidence Interval
CIBERESP	Centro de Investigación Biomédica en Red de Epidemiología y Salud Pública
COVID-19	Coronavirus Disease 2019
DL	DerSimonian–Laird estimator
DHI(s)	Digital Health Intervention(s)
DM2/T2DM	Type 2 Diabetes Mellitus
eHealth	Electronic Health
HbA1c	Glycated Hemoglobin
HTN	Hypertension
I^2^	Higgins’ I-squared statistic
IoT	Internet of Things
JMIR	Journal of Medical Internet Research
MD	Mean Difference
mHealth	Mobile Health
OSF	Open Science Framework
PRISMA	Preferred Reporting Items for Systematic Reviews and Meta-Analyses
RCT(s)	Randomized Controlled Trial(s)
REML	Restricted Maximum Likelihood
SE	Standard Error
SMS	Short Message Service
TIDieR	Template for Intervention Description and Replication
USA	United States of America
WHO	World Health Organization

## Appendix A

**Table A1:** Overview of study characteristics and participants.

Authors	Country	Sample (N)	Women (*n*)	Men (*n*)	Mean Age (Years)	HbA1c Inclusion Criteria
Daniel J Amante et al., 2021 [17]	USA	96	63	85	56.7	NR
Enying Gong et al., 2020 [23]	Australia	187	78	109	57	NR
Yeojin Kim et al., 2021 [24]	South Korea	68	38	30	57.2	NR
Shu-Fang Xia et al., 2022 [18]	China	120	43	77	56.7	≥7.0%
Kaisang Lin et al., 2022 [25]	China	50	27	23	53.3	≥7.0%
Zhiwei Lu et al., 2020 [26]	China	120	54	66	54.9	NR
Ramallo-Fariña et al., 2020 [27]	Spain Group 3 (only)	586	300	286	55.2	NR
LeChauncy Woodard et al., 2023 [28]	USA	280	16	264	67.2	>8.0%
Chen-Yu Han et al., 2023 [19]	China	418	308	110	51.95	NR
Mengna Guo et al., 2021 [20]	China	64	25	39	57.3	NR
Weiping Jia et al., 2021 [21]	USA	17,554	10322	7232	60.5	≥7.0%
Yuheng Feng et al., 2023 [29]	USA	225	116	109	65.6	>7.0%
Ryotaro Bouchi et al., 2024 [22]	Japan	1159	256	903	56.1	6.0–8.9%

Abbreviations: NR, Not reported.

**Table A2:** Selected characteristics for the study of subgroups of digital interventions using Tidier tool.

Authors	Essential Components of the Intervention	Materials/Procedures	Duration	Use of Glucometer	Intervention Provider	Duration	Intensity	Sessions and Duration	Timing of Outcomes
Daniel J Amante et al., 2021 [17]	Real-time monitoring of SMBG data for poorly controlled Type 2 Diabetes	Cellular-enabled glucose meter for automatic SMBG uploads	6 months	Yes	No	6 months	High	Access to on-demand telephone coaching	Baseline 6 months (each arm)
Enying Gong et al., 2020 [23]	Diabetes coaching using feedback and reinforcement via app-based system	My Diabetes Coach app with conversational agent to track HbA1c and HRQoL	12 months	No	No	12 months	Moderate	Weekly sessions or appointments	Baseline 6 months 12 months
Yeojin Kim et al., 2021 [24]	Self-management intervention based on the Information–Motivation–Behavioral skills model	IMB Model—participants used a smartphone app for weekly data input	6 months principal + 1 month following	No	No	6 months principal + 1 month following	Low	Not reported	Baseline 6 months
Shu-Fang Xia et al., 2022 [18]	Self-management guidance for diabetes via software	TangPlan, WeChat SMBG, and health education self-management guidance, with data recorded via WeChat	6 months	Yes	Multidisciplinary team of healthcare professionals	6 months	Moderate	Access to on-demand	Baseline 6 months
Kaisang Lin et al., 2022 [25]	Monitoring and management of metabolic indicators in T2DM patients via IoT	IoT technology-based diabetes management system: follow-up visits using IoT for patient data collection	3 months	No	No	3 months	Low	Not reported	Baseline 3 months
Zhiwei Lu et al., 2020 [26]	Effect of telemedicine patient management on blood glucose control in Chinese patients with T2DM	Patients received in-hospital pharmacy consultations and follow-up via the online platform	6 months	No	No	6 months	Low	Access to on-demand	Baseline 6 months
Ramallo-Fariña et al., 2020 [27]	Use of ICT to support decision-making in T2DM patients	Digital interventions for patients, professionals, or both	Over 24 months	No	Physicians, nurses, primary care professionals	Over 24 months	Moderate	8 face-to-face group sessions one every 3 months	Baseline 3 months 6 months 12 months 24 months
LeChauncy Woodard et al., 2023 [28]	Communication and self-management support for diabetes patients	RE-AIM model	10 months	No	Physicians, nurse educators, dietitians, and psychologists	10 months	Moderate	6 group sessions	Baseline 6 months post-intervention
Chen-Yu Han et al., 2023 [19]	Telemedicine for structured self-monitoring of blood glucose	SMBG	12 months	Yes	No	12 months	Low	Individual coaching on demand (needs-based tele-coaching)	Baseline 3 months 6 months post- intervention
Mengna Guo et al., 2021 [20]	mHealth management with an implantable glucose sensor for T2DM patients	Glucose sensor and mobile app mHealth management	1 month	Yes	General practitioners	1 month	Low	Not reported	Baseline 1 month
Weiping Jia et al., 2021 [21]	Mobile health (mHealth) intervention for better diabetes control in primary care	ROADMAP app and digital platform	12 months	Yes	Primary care doctors	12 months	High	At least 2 sessions each month	Baseline 12 months
Yuheng Feng et al., 2023 [29]	eHealth family intervention for T2DM patients based on family support	WeChat app modules and eHealth family-based intervention	12 months	No	Professional healthcare providers	12 months	Moderate	Not reported	Baseline 12 months
Ryotaro Bouchi et al., 2024 [22]	IoT-based approach to support lifestyle changes and improve diabetes care	IoT devices and mobile app IoT devices for self-monitoring and data submission	12 months	Yes	Doctors	12 months	High	Automatic feedback	Baseline 12 months

Abbreviations: SMBG: self-monitoring of blood glucose; HbA1c: glycated hemoglobin; T2DM: Type 2 Diabetes Mellitus; HRQoL: health-related quality of life; IoT: Internet of Things; ICTs: information and communication technologies; mHealth: mobile health; RE-AIM Model: reach, effectiveness, adoption, implementation, and maintenance.
