# Supplementary material for: Characteristics of Digital Health Interventions Associated with Improved Glycemic Control in T2DM: A Systematic Review and Meta-Analysis
Source: J Clin Med. 2026 Jan 31;15(3):1123. doi: 10.3390/jcm15031123 (PMC12897923; doi:10.3390/jcm15031123)
Supplement: Supplementary file 1 [file jcm-15-01123-s001.zip › jcm-4064814-supplementary.pdf]

SUPPLEMENTARY MATERIALS

S1.- PRISMA CHECKLIST

Section and Topic	Item #	Checklist item	Location where item is reported
**TITLE**			
Title	1	Identify the report as a systematic review.	Title p.1
**ABSTRACT**			
Abstract	2	See the PRISMA 2020 for Abstracts checklist.	Abstract p.1
**INTRODUCTION**			
Rationale	3	Describe the rationale for the review in the context of existing knowledge.	Introduction pp. 2-3
Objectives	4	Provide an explicit statement of the objective(s) or question(s) the review addresses.	Introduction p.3
**METHODS**			
Eligibility criteria	5	Specify the inclusion and exclusion criteria for the review and how studies were grouped for the syntheses.	Materials and Methods – Eligibility Criteria pp.3-4
Information sources	6	Specify all databases, registers, websites, organisations, reference lists and other sources searched or consulted to identify studies. Specify the date when each source was last searched or consulted.	Materials and Methods – Information Sources p.4
Search strategy	7	Present the full search strategies for all databases, registers and websites, including any filters and limits used.	Materials and Methods – Search Strategy; Supplementary Material S2 p.4
Selection process	8	Specify the methods used to decide whether a study met the inclusion criteria of the review, including how many reviewers screened each record and each report retrieved, whether they worked independently, and if applicable, details of automation tools used in the process.	Materials and Methods – Study Selection p.4
Data collection process	9	Specify the methods used to collect data from reports, including how many reviewers collected data from each report, whether they worked independently, any processes for obtaining or confirming data from study investigators, and if applicable, details of automation tools used in the process.	Materials and Methods – Data Collection Process p.5
Data items	10a	List and define all outcomes for which data were sought. Specify whether all results that were compatible with each outcome domain in each study were sought (e.g. for all measures, time points, analyses), and if not, the methods used to decide which results to collect.	Materials and Methods – Data Items pp. 5-6
	10b	List and define all other variables for which data were sought (e.g. participant and intervention characteristics, funding sources). Describe any assumptions made about any missing or unclear information.	Materials and Methods – Data Items pp. 5-6
Study risk of bias assessment	11	Specify the methods used to assess risk of bias in the included studies, including details of the tool(s) used, how many reviewers assessed each study and whether they worked independently, and if applicable, details of automation tools used in the process.	Materials and Methods – Risk of Bias Assessment; Supplementary Material S3 p.6

Section and Topic	Item #	Checklist item	Location where item is reported
Effect measures	12	Specify for each outcome the effect measure(s) (e.g. risk ratio, mean difference) used in the synthesis or presentation of results.	Materials and Methods – Data Analysis p.6
Synthesis methods	13a	Describe the processes used to decide which studies were eligible for each synthesis (e.g. tabulating the study intervention characteristics and comparing against the planned groups for each synthesis (item #5)).	Materials and Methods – Data Synthesis p.6
	13b	Describe any methods required to prepare the data for presentation or synthesis, such as handling of missing summary statistics, or data conversions.	Materials and Methods – Data Analysis pp.6-7
	13c	Describe any methods used to tabulate or visually display results of individual studies and syntheses.	Results – Figures 2–3; Tables A1–A2 pp. 8-12
	13d	Describe any methods used to synthesize results and provide a rationale for the choice(s). If meta-analysis was performed, describe the model(s), method(s) to identify the presence and extent of statistical heterogeneity, and software package(s) used.	Materials and Methods – Data Analysis pp. 6-7
	13e	Describe any methods used to explore possible causes of heterogeneity among study results (e.g. subgroup analysis, meta-regression).	Materials and Methods – Subgroup Analysis; Meta-Regression pp. 7-8
	13f	Describe any sensitivity analyses conducted to assess robustness of the synthesized results.	Materials and Methods – Sensitivity Analyses p.7
Reporting bias assessment	14	Describe any methods used to assess risk of bias due to missing results in a synthesis (arising from reporting biases).	Materials and Methods – Trim-and-Fill Analysis p.7
Certainty assessment	15	Describe any methods used to assess certainty (or confidence) in the body of evidence for an outcome.	Certainty of evidence assessed using the GRADE approach; Supplementary Materials (GRADE Summary of Findings)
**RESULTS**			
Study selection	16a	Describe the results of the search and selection process, from the number of records identified in the search to the number of studies included in the review, ideally using a flow diagram.	Results – Study Selection; p.8 Figure 1
	16b	Cite studies that might appear to meet the inclusion criteria, but which were excluded, and explain why they were excluded.	Results – Study Selection p.8
Study characteristics	17	Cite each included study and present its characteristics.	Results – Study

Section and Topic	Item #	Checklist item	Location where item is reported
			Characteristics; Table A1 pp.8-9
Risk of bias in studies	18	Present assessments of risk of bias for each included study.	Results – Risk of Bias pp. 9-10
Results of individual studies	19	For all outcomes, present, for each study: (a) summary statistics for each group (where appropriate) and (b) an effect estimate and its precision (e.g. confidence/credible interval), ideally using structured tables or plots.	Results – Meta-analysis; Figure 2 pp. 10-11
Results of syntheses	20a	For each synthesis, briefly summarise the characteristics and risk of bias among contributing studies.	Results – Meta-analysis
	20b	Present results of all statistical syntheses conducted. If meta-analysis was done, present for each the summary estimate and its precision (e.g. confidence/credible interval) and measures of statistical heterogeneity. If comparing groups, describe the direction of the effect.	Results – Meta-analysis pp. 10-12
	20c	Present results of all investigations of possible causes of heterogeneity among study results.	Results – Subgroup Analysis; Meta-Regression pp. 11 -12
	20d	Present results of all sensitivity analyses conducted to assess the robustness of the synthesized results.	Results – Sensitivity Analyses p.12
Reporting biases	21	Present assessments of risk of bias due to missing results (arising from reporting biases) for each synthesis assessed.	Results – Trim-and-Fill Analysis p.12
Certainty of evidence	22	Present assessments of certainty (or confidence) in the body of evidence for each outcome assessed.	Certainty of evidence presented using GRADE; Supplementary Materials
**DISCUSSION**			
Discussion	23a	Provide a general interpretation of the results in the context of other evidence.	Discussion pp.13-14
	23b	Discuss any limitations of the evidence included in the review.	Discussion – Strengths and Limitations pp. 14-15
	23c	Discuss any limitations of the review processes used.	Discussion – Strengths and Limitations p.15
	23d	Discuss implications of the results for practice, policy, and future research.	Discussion pp. 15-16
**OTHER INFORMATION**			
Registration and protocol	24a	Provide registration information for the review, including register name and registration number, or state that the review was not registered.	Materials and Methods – Registration (OSF) p.3
	24b	Indicate where the review protocol can be accessed, or state that	Materials and

Section and Topic	Item #	Checklist item	Location where item is reported
		a protocol was not prepared.	Methods – Registration (OSF) p.3
	24c	Describe and explain any amendments to information provided at registration or in the protocol.	Not applicable
Support	25	Describe sources of financial or non-financial support for the review, and the role of the funders or sponsors in the review.	Funding p.16
Competing interests	26	Declare any competing interests of review authors.	Conflicts of Interest p.16
Availability of data, code and other materials	27	Report which of the following are publicly available and where they can be found: template data collection forms; data extracted from included studies; data used for all analyses; analytic code; any other materials used in the review.	Supplementary Materials p.16

From: Page MJ, McKenzie JE, Bossuyt PM, Boutron I, Hoffmann TC, Mulrow CD, et al. The PRISMA 2020 statement: an updated guideline for reporting systematic reviews. BMJ 2021;372:n71. doi: 10.1136/bmj.n71. This work is licensed under CC BY 4.0. To view a copy of this license, visit https://creativecommons.org/licenses/by/4.0/

S2.- SEARCHING STRATEGY

Database	Enhanced Search Strategy
PubMed	(("type 2 diabetes mellitus"[MeSH Terms] OR "diabetes mellitus type 2"[Title/Abstract] OR "type 2 diabetes"[Title/Abstract] OR "type II diabetes mellitus"[Title/Abstract] OR "DM2"[Title/Abstract] OR "non insulin dependent diabetes"[Title/Abstract] OR "adult onset diabetes"[Title/Abstract] OR "maturity onset diabetes"[Title/Abstract]) AND ("mobile health"[MeSH Terms] OR "mHealth"[Title/Abstract] OR "m-health"[Title/Abstract] OR "mobile application"[Title/Abstract] OR "digital app"[Title/Abstract] OR "eHealth"[Title/Abstract] OR "digital intervention"[Title/Abstract]) OR “telemonitoring interventions” OR “mobile apps interventions” OR “SMS interventions” OR “Web-based plataforms intervention” AND ("clinical trial"[Publication Type] OR "randomized controlled trial"[Publication Type] OR "RCT"[Title/Abstract]) AND ("HbA1c"[Title/Abstract] OR "glycosylated hemoglobin"[Title/Abstract] OR "glycated hemoglobin"[Title/Abstract] OR "hemoglobin A1c"[MeSH Terms]) AND ("effectiveness"[Title/Abstract] OR "efficacy"[Title/Abstract] OR "treatment outcome"[MeSH Terms]))
Embase	(('type 2 diabetes mellitus'/exp OR 'diabetes mellitus type 2':ti,ab OR 'type II diabetes mellitus':ti,ab OR 'DM2':ti,ab OR 'non insulin dependent diabetes':ti,ab OR 'adult onset diabetes':ti,ab OR 'maturity onset diabetes':ti,ab) AND ('mobile health'/exp OR 'mHealth':ti,ab OR 'm-health':ti,ab OR 'digital app':ti,ab OR 'eHealth':ti,ab OR 'mobile application':ti,ab OR 'digital intervention':ti,ab OR 'telemonitoring

	interventions':ti,ab OR 'mobile apps interventions':ti,ab OR 'SMS interventions':ti,ab OR 'Web-based plataforms intervention':ti,ab) AND<br>('randomized controlled trial'/exp OR 'clinical trial':ti,ab OR 'RCT':ti,ab) AND<br>('hemoglobin A1c'/exp OR 'glycosylated hemoglobin':ti,ab OR 'HbA1c':ti,ab OR 'glycated hemoglobin':ti,ab) AND<br>('effectiveness':ti,ab OR 'efficacy':ti,ab OR 'treatment outcome'/exp))
Cochrane Library	(("type 2 diabetes" OR "type II diabetes mellitus" OR "diabetes mellitus type 2" OR "DM2" OR<br>"non insulin dependent diabetes" OR "adult onset diabetes" OR "maturity onset diabetes") AND<br>("mobile health" OR "mHealth" OR "digital app" OR "mobile application" OR "eHealth" OR<br>"digital intervention" OR “telemonitoring interventions” OR “mobile apps interventions” OR “SMS interventions” OR “Web-based plataforms intervention”) AND<br>("clinical trial" OR "randomized controlled trial" OR "RCT") AND<br>("HbA1c" OR "hemoglobin A1c" OR "glycosylated hemoglobin" OR "glycated hemoglobin") AND<br>("effectiveness" OR "efficacy" OR "treatment outcome"))
JMIR	(("type 2 diabetes" OR "type II diabetes" OR "diabetes mellitus type 2" OR "DM2" OR<br>"non insulin dependent diabetes" OR "adult onset diabetes" OR "maturity onset diabetes") AND<br>("digital app" OR "mHealth" OR "mobile health" OR "eHealth" OR "digital intervention" OR "mobile application" OR “telemonitoring interventions” OR “mobile apps interventions” OR “SMS interventions” OR “Web-based plataforms intervention”) AND<br>("HbA1c" OR "glycosylated hemoglobin" OR "glycated hemoglobin" OR "hemoglobin A1c") AND<br>("clinical trial" OR "RCT" OR "randomized controlled trial") AND<br>("effectiveness" OR "efficacy" OR "treatment outcome"))

S3.- TEMPLATE FOR THE CHECKLIST FOR THE DESCRIPTION AND REPLICATION OF INTERVENTIONS (TIDIER)

Key: R08

1	Name of intervention	Livongo
2	Describe the theoretical framework or the purpose of the essential components of the intervention	Patients with poorly controlled type 2 diabetes (T2D) experience increased morbidity, increased mortality, and higher cost of care. Self-monitoring of blood glucose (SMBG) is a critical component of diabetes self-management with established diabetes outcome benefits. Technological advancements in blood glucose meters, including cellular-connected devices that automatically upload SMBG data to secure cloud-based databases, allow for improved sharing and

		monitoring of SMBG data. Real-time monitoring of SMBG data presents opportunities to provide timely support to patients that is responsive to abnormal SMBG recordings. Such diabetes remote monitoring programs can provide patients with poorly controlled T2D additional support needed to improve critical outcomes.
3	Materials	Livongo Diabetes Program, is accredited by the American Association of Diabetes Educators (AADE) Diabetes Education Accreditation Program and includes access to both scheduled and in-the-moment CDE support via phone call or SMS text messaging. At the time of the study, the Livongo for Diabetes program was not available as a direct-to-consumer product but was available to employees of several large companies.
4	Procedures	The In Touch connected glucose meter is cellular-enabled, allowing for automatic uploading of self-monitoring of blood glucose (SMBG) recordings to a secure patient portal. Patients were instructed to use the meter to test their blood glucose as frequently as previously instructed by their providers. After patients use the meter to test their glucose, the SMBG recording is uploaded to the Livongo Smart Cloud. In this study, Livongo transferred all SMBG data to the DCOE electronic health record (EHR) system daily.
5	Describe the experience, training, and qualifications of the individual delivering the intervention	NA
6	Mode of intervention delivery	Free recruitment of people who wish to participate in the project for a period of 6 months.
7	Setting where the intervention is delivered	United States
8	Describe the number of times the intervention was delivered and over what period, including the number of sessions, timing, duration, and intensity or dose	It was carried out over a period of 6 months with daily sending of glucose measurements to the Livongo Smart Cloud, while the Livongo CDE Care Team makes weekly reports to the Diabetes center of excellence to which the patients belong.
9	If the intervention was planned to be personalized, tailored, or adapted, describe what was adapted, why, when, and how	No, it is a general application for all patients.
10	If the intervention was modified during the course of the study, describe the changes	It was not modified

	(what, why, when, and how)	
11	If adherence to or fidelity of the intervention was assessed, describe how and by whom it was evaluated	Yes, through the Livongo CDE Care Team
12	If adherence to or fidelity to the intervention was assessed, describe the extent to which it was delivered as planned.	NA

Key: R12

1	Name of intervention	My Diabetes Coach
2	Describe the theoretical framework or the purpose of the essential components of the intervention	Diabetes coaching programs that incorporate feedback and reinforcement have proven to be an effective strategy to improve the management of glucose levels.
3	Materials	My Diabetes Coach (MDC) program, an app-based interactive embodied conversational agent, Laura, designed to support diabetes self-management in the home.
4	Procedures	This randomized controlled trial evaluated both the implementation and effectiveness of the MDC program. Adults with type 2 diabetes in Australia were recruited and randomized to the intervention arm (MDC) or the control arm (usual care).<br>Program use was tracked over 12 months. Coprimary outcomes included changes in glycated hemoglobin (HbA1c) and health-related quality of life (HRQoL). Data were assessed at baseline and at 6 and 12 months, and analyzed using linear mixed-effects regression models.
5	Describe the experience, training, and qualifications of the individual delivering the intervention	NA
6	Mode of intervention delivery	Participants were encouraged to use the app weekly to complete online modules by chatting with Laura or touching buttons on the screen. Each appointment module with Laura began with a review of progress with feedback, education and counseling on the chosen topic, and incorporated tips on overcoming barriers, followed by a short quiz and closing remarks

7	Setting where the intervention is delivered	Of the 697 individuals with T2DM who expressed interest in participating in the study, 187 were recruited, including 62 (33%) from Victoria, 21 (16.5%) from New South Wales, and 21 (16.5%) from Queensland
8	Describe the number of times the intervention was delivered and over what period, including the number of sessions, timing, duration, and intensity or dose	12 months, 2 periods: 6 and 12 months.
9	If the intervention was planned to be personalized, tailored, or adapted, describe what was adapted, why, when, and how	No changes were made.
10	If the intervention was modified during the course of the study, describe the changes (what, why, when, and how)	NA
11	If adherence to or fidelity of the intervention was assessed, describe how and by whom it was evaluated	Program effectiveness was measured by both clinical and psycho-behavioral outcomes. The coprimary outcomes were changes (12 months compared with baseline) in glycated hemoglobin (HbA1c) and health-related quality of life (HRQoL), which were examined in terms of between-arm differences. The secondary time point of analysis examined the change between baseline and 6 months. HbA1c (reported as % and mmol/mol) was measured through a pathology blood test that each participant's GP requested. HRQoL was assessed via participants' completion of the Assessment of Quality of Life (AQoL)-8D scale, which is a 35-item multi-attribute utility instrument covering 8 dimensions focused on independent living, happiness, mental health, coping, relationships, self-worth, pain, and senses.
12	If adherence to or fidelity to the intervention was assessed, describe the extent to which it was delivered as planned.	According to the plans.

Key: R15

1	Name of intervention	The IMB Model

2	Describe the theoretical framework or the purpose of the essential components of the intervention	The global prevalence rate of diabetic adults in 2000 was 115 million, which tripled to 466 million in 2019 and continues to increase. It is expected to reach 578 million by 2030, with an estimated 700 million by 2045. The prevention of diabetic complications requires not only medication but also self-management, such as diet, exercise, and monitoring of blood sugar levels. Recently, self-management interventions for people with diabetes have focused on changing individuals' perceptions, such as attitudes or beliefs toward behavior and psychosocial coping skills that could ultimately lead to changes in self-management behavior. Many previous studies have applied the Information-Motivation-Behavioral skills (IMB) model to explain the self-management behaviors of people with diabetes and have confirmed the model's compatibility.
3	Materials	Information-Motivation-Behavioral skills (IMB) Model
4	Procedures	The G-Power Analysis software program (G-power 3.1.9) was used to calculate the number of participants required for this study. The inclusion criteria were those aged 19 years and older, with HbA1c over 6.5%, taking diabetes or insulin medication for more than six months, able to walk, can communicate in and read Korean, were using the smartphone app with data input at least once a week, and those using the phone operating systems Android 4.3 or iOS 5.1 or higher.
5	Describe the experience, training, and qualifications of the individual delivering the intervention	NA
6	Mode of intervention delivery	A total of 4,160 patients visited the university hospital's outpatient endocrinology department during that time, and 2,404 of those patients met the study criteria. However, 2,332 people were excluded due to personal circumstances, with reasons such as not being motivated to participate in the research, difficulty in participating due to work, not wanting to use the app, or difficulty in completing the survey. In total, 72 patients were numbered in the order in which they participated in the study and signed the consent form.
7	Setting where the intervention is delivered	Pusan National University Hospital, Busan, South Korea
8	Describe the number of times the intervention was delivered and over what period, including the number of sessions, timing, duration, and intensity or dose	This study took place from March 2 to September 20, 2019, and was conducted by the researcher and four research assistants.<br>The researchers provided intervention, one research assistant made random assignments using random number generators, two assisted with data collection and smartphone app user training, and the fourth performed data coding and

		analysis after data collection. The two research assistants who helped in data collection were outpatient endocrinology nurses, each with two or more years of clinical experience in endocrinology. The researcher educated them about the study's contents and objectives, the composition of the questionnaire, and the app usage.
9	If the intervention was planned to be personalized, tailored, or adapted, describe what was adapted, why, when, and how	Not
10	If the intervention was modified during the course of the study, describe the changes (what, why, when, and how)	Not
11	If adherence to or fidelity of the intervention was assessed, describe how and by whom it was evaluated	NA
12	If adherence to or fidelity to the intervention was assessed, describe the extent to which it was delivered as planned.	NA

Key: R18

1	Name of intervention	TangPlan and WeChat
2	Describe the theoretical framework or the purpose of the essential components of the intervention	China has the largest number of patients with type 2 diabetes mellitus (T2DM) in the world. However, owing to insufficient knowledge of self-management in patients with diabetes, blood glucose (BG) control is poor. Most diabetes-related self-management applications fail to bring significant benefits to patients with T2DM because of the low use rate and difficult operation.
3	Materials	Software TangPlan and WeChat
4	Procedures	Participants were recruited and randomized into the TangPlan and WeChat or control groups. Participants in the control group received usual care, whereas the TangPlan and WeChat participants received self-management guidance with the help of TangPlan and WeChat from health care professionals, including BG self-monitoring; healthy eating; active physical exercise; increasing medication compliance; and health

		education during follow-ups, lectures, or web-based communication. They were also asked to record and send self-management data to the health care professionals via WeChat to obtain timely and effective guidance on diabetes self-management.
5	Describe the experience, training, and qualifications of the individual delivering the intervention	Multidisciplinary team comprised health care professionals, including the general physician from the community health care center, a diabetes specialist nurse, physicians from the department of endocrinology, physicians from the department of rehabilitation, a dietitian, and trained diabetes health educators.
6	Mode of intervention delivery	A total of 343 participants with T2DM were assessed for eligibility, of whom 187 (54.5%) were excluded. In total, 52 participants declined to take part because they were not interested in the program (18/52, 35%), did not want to pay much attention to diabetes (14/52, 27%), thought they did not need help (15/52, 29%), or had no reason (5/52, 10%). A total of 156 participants were randomized into the TangPlan and WeChat group (78/156, 50%) or the control group (78/156, 50%). Of these, 120 participants (TangPlan and WeChat: 64/120, 53.3%; control: 56/120, 46.7%) completed the follow-up assessments, yielding a retention rate of 76.9% (120/156).
7	Setting where the intervention is delivered	Affiliated Hospital of Jiangnan University, Wuxi, China.
8	Describe the number of times the intervention was delivered and over what period, including the number of sessions, timing, duration, and intensity or dose	One time. Over a 6-months period.
9	If the intervention was planned to be personalized, tailored, or adapted, describe what was adapted, why, when, and how	Not
10	If the intervention was modified during the course of the study, describe the changes (what, why, when, and how)	Not
11	If adherence to or fidelity of the intervention was assessed, describe how	NA

	and by whom it was evaluated	
12	If adherence to or fidelity to the intervention was assessed, describe the extent to which it was delivered as planned.	NA

Key: R21

1	Name of intervention	NA
2	Describe the theoretical framework or the purpose of the essential components of the intervention	Type 2 diabetes (T2DM) is a complex metabolic disease. Apart from pathoglycemia, T2DM occurs with the abnormalities of some metabolic indexes, including weight, blood pressure, and blood fat. Epidemiological investigation demonstrates that 113.9 million people among Chinese adults suffer from diabetes and its complications.
3	Materials	The information about the research objects was as follows: a total of 50 T2DM patients receiving diabetes treatment in hospital between January 2019 and July 2020 was selected as the research objects. All selected cases were randomly divided into control group and joint group, each of which included 25 cases. Patients in two groups were all monitored by IoT technology-based diabetes management information system.
4	Procedures	IoT technology-based diabetes management information system was utilized for follow-up visits. Based on the observation of weight and height values before and after treatment, BMI values were calculated.<br>Systolic blood pressure (SBP) and diastolic blood pressure (DBP) were measured. Besides, early morning fasting venous blood in the early morning and 2 hours after breakfast were extracted to detect fasting plasma glucose (FPG)(mmoL/L), fasting insulin (FINS) (μU/mL), 2 h postprandial blood glucose (2hPG), glycosylated hemoglobin (HbAlc) (%), total cholesterol (TC), triglyceride (TG), and low density lipoprotein (LDL).
5	Describe the experience, training, and qualifications of the individual delivering the intervention	NA
6	Mode of intervention delivery	To explore the application value of medical intelligent electronic system under the background of Internet of things in the clinical study of the treatment of overweight/obesity in type 2 diabetes mellitus (T2DM) with empagliflozin combined with liraglutide; 50 overweight and obese adult T2DM patients

		in our hospital were randomly divided into the combined group and the<br>control group, 25 cases in each group. The control group was treated with liraglutide alone, while the combined group was treated with empagliflozin on the basis of liraglutide. Based on the Internet of things technology, with diabetes management as the core, the functions of information collection, transmission, and storage of T2DM patients are realized. Doctors pass the diabetes management plan to T2DM patients through the platform, supervise the implementation, and finally compare the clinical efficacy of the two groups.
7	Setting where the intervention is delivered	The third affiliated Hospital of Southern Medical University, China
8	Describe the number of times the intervention was delivered and over what period, including the number of sessions, timing, duration, and intensity or dose	Only once. 3 months.
9	If the intervention was planned to be personalized, tailored, or adapted, describe what was adapted, why, when, and how	Not
10	If the intervention was modified during the course of the study, describe the changes (what, why, when, and how)	Not
11	If adherence to or fidelity of the intervention was assessed, describe how and by whom it was evaluated	NA
12	If adherence to or fidelity to the intervention was assessed, describe the extent to which it was delivered as planned.	NA

Key: R22

1	Name of intervention	NA

2	Describe the theoretical framework or the purpose of the essential components of the intervention	Effect of telemedicine patient management on blood glucose control in Chinese patients with T2DM<br>Internet-based medication management services might help improve blood glucose control in patients with diabetes.
3	Materials	This study included patients with T2DM from the Department of Integrated Traditional Chinese and Western medicine of Tianjin Medical University Chu Hsien-I Memorial Hospital who consulted between January 2019 and December 2019; 120 patients were randomized to the control group (standard care) and the telemedicine group (in-hospital medication evaluation, drug reorganization, medical monitoring, and advice via telemedicine). The primary endpoint was targeted glycated hemoglobin (HbA1c) <7.0% from baseline to 6 months. The secondary endpoints were the changes in fasting blood glucose (FBG), changes in daily medication cost, changes in the number of drug types taken daily, and hypoglycemic events.
4	Procedures	Patients received in-hospital pharmacy consultations and follow-up via the online platform
5	Describe the experience, training, and qualifications of the individual delivering the intervention	Not mentioned
6	Mode of intervention delivery	This study is a randomized controlled trial designed to explore the effect of telemedicine patient management on blood glucose control in Chinese patients with type 2 diabetes mellitus (T2DM). The sample size was 120 cases randomized by using a 1:1 matching design. The patients were randomized into two groups, an intervention and a control group, by using a random number table. An open-label method was used (patients and providers could not be blinded to the interventions). At admission, according to the pre-established random number table, the staff who did not participate in the collection of the study data informed the investigators of the assigned group for each patient, with 60 patients in each group.
7	Setting where the intervention is delivered	Memorial Hospital, Tianjin, China.
8	Describe the number of times the intervention was delivered and over what period, including the number of sessions, timing, duration, and intensity or dose	Only once.<br>Control group. The patients in the control group received standard care according to the hospital's standard procedures.<br>The patients in the telemedicine group first received an inhospital pharmacy consultation and medication evaluation, and they then underwent drug reorganization with medical monitoring and advice.<br>The participants in the control group visited the outpatient

		clinics every 2 weeks from 14 days after discharge. They carried out daily self-blood glucose monitoring at home. HbA1c was not measured during follow-up, only at the end of the 6-month follow-up. There was no tracking, only patient self-monitoring.<br>In-hospital pharmacy consultation, medication evaluation, and treatment. Pharmacists used a mobile clinical pharmacy software to browse the electronic medical records and in-patient medical orders and conducted face-to-face pharmacy consultations for each patient. This involved collecting and recording basic information on patients' medications, including basic conditions, current symptoms and physical examination, past medical history, operation history, personal history, allergic history, past medication history, related examinations,<br>current medication, and laboratory test results.<br>In general, for the telemedicine group. The follow-up is through the internet. Patients came to the hospital outpatient service to obtain medicines every month or when the treatment plan was adjusted, and a new prescription was required. All other follow-ups were through the online pharmacy platform. HbA1c was not evaluated during follow-up, only at the end of follow-up. Fasting and 2-h postprandial blood glucose levels were tested by the patients themselves. The patients could transmit the data measured by the blood glucose monitors to the WeChat applet via Bluetooth and send it to the pharmacist service platform.
9	If the intervention was planned to be personalized, tailored, or adapted, describe what was adapted, why, when, and how	Not
10	If the intervention was modified during the course of the study, describe the changes (what, why, when, and how)	Not
11	If adherence to or fidelity of the intervention was assessed, describe how and by whom it was evaluated	NA
12	If adherence to or fidelity to the intervention was assessed, describe the	NA

	extent to which it was delivered as planned.	

Clave: R23

1	Name of intervention	Indica STUDY
2	Describe the theoretical framework or the purpose of the essential components of the intervention	Type 2 diabetes mellitus (T2DM) is a chronic condition in which long-term health outcomes are related to patients' adherence to lifestyle modifications and pharmacologic treatments. Other stakeholders, such as relatives and primary health care professionals, are also involved in guiding patients' decisions. To address these unmet needs, many publications have reported on the effectiveness of using information and communications<br>technology (ICT) applications to support decision making by patients and professionals [8-12], reporting favorable short-term effects on blood glucose control [11,12]. The effectiveness of other biological, cognitive, behavioral, or emotional outcome measures remains controversial.
3	Materials	INDICA's Intervention<br>It's not described the content.
4	Procedures	
5	Describe the experience, training, and qualifications of the individual delivering the intervention	Physicians, nurses and primary care professionals.
6	Mode of intervention delivery	The INDICA study is an open, community-based pragmatic, multicenter, clinical controlled trial with random allocation by clusters to usual care or to one of the following 3 interventions of knowledge transfer and behavior modification:<br>• Group 1 included interventions for patients and a family member (intervention for patients)<br>• Group 2 included interventions for health care professionals (physicians and nurses) at primary care (intervention for professionals)<br>• Group 3 combined the interventions for patients and professionals (combined intervention)
7	Setting where the intervention is delivered	PHCPs were recruited in 4 Canary Islands (Tenerife, Gran Canaria, Lanzarote, and La Palma).
8	Describe the number of times the intervention was delivered and over what period, including the number of sessions,	One.<br>In 3 groups, previously described.<br>Follow for 24 months.<br>Group 1: 8 digital interventions. At the beginning of the intervention and one each 3 months.

	timing, duration, and intensity or dose	Group 2: 2 sessions for professionals, one at the beginning of the interventions and one after 3 months.<br>Group 3: 2 sessions for professionals, one at the beginning of the interventions and one after 3 months.<br>8 digital interventions. At the beginning of the intervention and one each 3 months.
9	If the intervention was planned to be personalized, tailored, or adapted, describe what was adapted, why, when, and how	Not
10	If the intervention was modified during the course of the study, describe the changes (what, why, when, and how)	Not
11	If adherence to or fidelity of the intervention was assessed, describe how and by whom it was evaluated	NA
12	If adherence to or fidelity to the intervention was assessed, describe the extent to which it was delivered as planned.	NA

Clave R: 24

1	Name of intervention	EPICC
2	Describe the theoretical framework or the purpose of the essential components of the intervention	Type 2 diabetes is a prevalent condition that contributes to adverse outcomes, such as stroke, kidney failure, blindness, and heart diseases. Guidelines for diabetes control, measured by hemoglobin A1c (HbA1c) levels, arise from clinical trials demonstrating lower morbidity and mortality with lowering of HbA1c levels. Because type 2 diabetes is a chronic condition, achieving control requires patient activation and commitment with treatment planning, medications, and self-management. Interventions facilitating communication and collaboration between patients and clinicians that support self-management have the potential for improving diabetes-associated distress and glycemic control.
3	Materials	We used RE-AIM (reach, effectiveness, adoption, implementation, and maintenance) to guide analyses for this study.

4	Procedures	It was developed a research-practice partnership with practice leaders and 20 nonacademic health care professionals (ie, dietitians, nurses, pharmacists, and physicians) who provided usual care from 3 hospital-based and 2 community-based primary care clinics in Illinois, Indiana, and Texas to deliver EPICC within routine care. Partnership building facilitated practice and partner recruitment, training and validation of clinicians in EPICC protocols, and implementation within care workflows.
5	Describe the experience, training, and qualifications of the individual delivering the intervention	Physicians, nurse educators, nurse practitioners, pharmacists, dietitians, and psychologists
6	Mode of intervention delivery	Participants in EPICC attended 6 group sessions based on a collaborative goalsetting theory led by health care professionals. Clinicians conducted individual motivational interviewing sessions after each group.<br>Usual care was enhanced (EUC) with diabetes education.
7	Setting where the intervention is delivered	Veterans Affairs clinics in Illinois, Indiana, and Texas.
8	Describe the number of times the intervention was delivered and over what period, including the number of sessions, timing, duration, and intensity or dose	Once. In 6 groups.<br>The study used a hybrid randomized trial design to evaluate EPICC effectiveness on diabetes outcomes in the context of implementation within 2 regional VA health systems.<br>3 months intervention, 6 months follow for maintenance period. EPICC participants attended 6 bimonthly group sessions (duration of approximately 1 hour) based on collaborative goal setting and motivational interviewing theory during a 3-month period.<br>3-hour training workshop prepared health care professionals (physicians, nurse educators, nurse practitioners, pharmacists, dietitians, and psychologists) to lead sessions and conduct 10-minute individual sessions immediately following group sessions with each participant.
9	If the intervention was planned to be personalized, tailored, or adapted, describe what was adapted, why, when, and how	Not
10	If the intervention was modified during the course of the study, describe the changes (what, why, when, and how)	Not

11	If adherence to or fidelity of the intervention was assessed, describe how and by whom it was evaluated	NA
12	If adherence to or fidelity to the intervention was assessed, describe the extent to which it was delivered as planned.	The primary outcome consisted of changes in HbA1c levels after the intervention and during maintenance. Secondary outcomes included the Diabetes Distress Scale (DDS), Morisky Medication Adherence Scale, and Lorig Self-efficacy Scale. Secondary implementation outcomes included reach, adoption, and implementation (number of sessions attended per patient).

Key: R27

1	Name of intervention	SINOMEDISITE
2	Describe the theoretical framework or the purpose of the essential components of the intervention	Diabetes mellitus (DM) is one of the world's most serious non-infectious diseases and a major threat to human health. The World Health Organization estimates that 366 million patients will be suffering from diabetes by 2030, twice the number of patients in 2000. The incidence and severity of complications mainly depend on the course of diabetes and the control of blood glucose. Therefore, good metabolic control is very important for patients with T2DM. Telemedicine is a very promising tool for delivering personalized SMBG and healthcare at home or where it is needed, reducing the unnecessary use of healthcare resources.
3	Materials	Thus, based on the premise that telemedicine-assisted structured SMBG may increase the efficacy of treatment and self-regulation, this study aimed to evaluate the effects of telemedicine assisted structured self-monitoring of blood glucose on glycemic control and diabetes management of Chinese patients.
4	Procedures	The participants were allocated randomly to an intervention group or a control group. The study was not blinded. Before the study, the patients in both groups received a self-management guide and a blood glucose meter (BGM). The name of the BGM was SINOMEDISITE.
5	Describe the experience, training, and qualifications of the individual delivering the intervention	NA
6	Mode of intervention delivery	The participants were allocated randomly to an intervention group or a control group. The study was not blinded.

7	Setting where the intervention is delivered	Department of Endocrinology, Qingpu Branch of Zhongshan Hospital affiliated to Fudan University
8	Describe the number of times the intervention was delivered and over what period, including the number of sessions, timing, duration, and intensity or dose	Once. 6 months.<br>For the patients in both groups, the following monitoring strategy was devised: The structured monitoring consisted of six points (before each meal and 2 h after eating) if three main meals were consumed daily and whenever there was a risk of hypoglycemia, especially at night.
9	If the intervention was planned to be personalized, tailored, or adapted, describe what was adapted, why, when, and how	Not
10	If the intervention was modified during the course of the study, describe the changes (what, why, when, and how)	Not
11	If adherence to or fidelity of the intervention was assessed, describe how and by whom it was evaluated	NA
12	If adherence to or fidelity to the intervention was assessed, describe the extent to which it was delivered as planned.	NA

Key: R28

1	Name of intervention	NA
2	Describe the theoretical framework or the purpose of the essential components of the intervention	With the improvement of Chinese people's living standards and changes in lifestyle, China now has the most adult diabetics in the world, and this is predicted to increase to 120 million by 2045 indicating a tremendous public health burden in China. Patients with type 2 diabetes mellitus (T2DM) account for the vast majority of those with diabetes.
3	Materials	This study aimed to evaluate the effectiveness of mHealth management with an implantable glucose sensor and a mobile application among patients with type 2 diabetes mellitus (T2DM) in China.

4	Procedures	A randomised controlled trial was carried out to compare the effectiveness of usual health management to mHealth management based on a model that consisted of the network platform, an implantable glucose sensor and a mobile app featuring guidance from general practitioners (GPs) over a four-week period.
5	Describe the experience, training, and qualifications of the individual delivering the intervention	Only described as general practitioners (GPs).
6	Mode of intervention delivery	This was a parallel-group, two-arm randomised controlled pilot trial to assess the feasibility and to compare the effectiveness of a four-week mHealth intervention to usual health management for patients with T2DM. The trial groups were parallel in the sense that members of each cohort received only the allocated management without any crossovers. The health management period for the two groups was four weeks.
7	Setting where the intervention is delivered	General hospital in Hangzhou City, Zhejiang Province, PR China.
8	Describe the number of times the intervention was delivered and over what period, including the number of sessions, timing, duration, and intensity or dose	Once. Four weeks.<br>Patients in the intervention group received mHealth management based on the mHealth management model that consisted of the network platform, an implantable glucose sensor, a mobile app and GP support.<br>First, the implantable glucose sensor was subcutaneously implanted to enable patients to monitor their blood glucose at any time. It could be used continuously for 14 days before the power ran out. The health management period was four weeks. So, on the 15th day, a second sensor was subcutaneously implanted and the first sensor was removed. The blood glucose values were automatically transmitted to the processor to produce a dynamic trend graph of the blood glucose values.<br>Second, both patients and GPs downloaded the mobile app. For patients, the app included four functional modules: (a) fill in the health information, (b) obtain real-time blood glucose values and a dynamic trend graph, (c) communicate with a GP and (d) obtain personalised intervention programmes. For GPs, the app included three functional modules: (a) obtain real-time information from patients and the processor, (b) fill in personalised intervention programmes for patients and (c) communicate with patients.
9	If the intervention was planned to be personalized, tailored, or adapted, describe what	Not

	was adapted, why, when, and how	
10	If the intervention was modified during the course of the study, describe the changes (what, why, when, and how)	Not
11	If adherence to or fidelity of the intervention was assessed, describe how and by whom it was evaluated	NA
12	If adherence to or fidelity to the intervention was assessed, describe the extent to which it was delivered as planned.	NA

Key: R29

1	Name of intervention	ROADMAP
2	Describe the theoretical framework or the purpose of the essential components of the intervention	An innovative mobile health (mHealth)-enabled hierarchical diabetes management intervention was introduced and evaluated in China with the purpose of achieving better control of type 2 diabetes in primary care.
3	Materials	A mobile health (mHealth)-based digital platform named Road to Hierarchical Diabetes Management at Primary Care Settings in China (ROADMAP) was designed and tested through a community-based cluster randomized controlled trial that covered 19,546 participants from 864 communities across China.
4	Procedures	The trial involved a total of 864 communities in 144 counties across 25 eligible provinces in mainland China, covering developed/less developed and urban/rural areas. Within each province, an average of 6 counties and 36 subordinate communities (6 communities from each county) participated in the trial. In each community (cluster), an average of 22 participants were selected at random from a full list of type 2 diabetes patients from local BPHS system.
5	Describe the experience, training, and qualifications of the individual delivering the intervention	Doctors from primary care.

6	Mode of intervention delivery	The multicomponent intervention contained a contracted service package, service providers–targeted capacity building, and performance review for service delivery, which was facilitated through a provider-facing smartphone application Graded ROADMAP and a website. The application connected doctors from primary care clinics within the communities and county hospitals and constituted the hierarchy of care team in the region. Contracted service package was a set of structured diabetes management services, initiated by blood glucose monitoring and BP measurement at each monthly clinic visit. Fingertip blood glucose monitoring was performed with a Graded ROADMAP–bundled unified meter.<br>A minimum of one fasting and one postprandial blood glucose (on the same day); one BP measurement; one diabetic peripheral neuropathy screening; diet and physical exercise consultation and medication instruction were suggested at each face-to-face visit. The primary care doctors kept track of the results of delivered services with Graded ROADMAP app and took on a role alike gatekeeper by proactively providing patients with routine contacts, monitoring and evaluation, and lifestyle instructions. The app would remind the primary care doctor to request a referral when it captured an indication in patient's record.
7	Setting where the intervention is delivered	China
8	Describe the number of times the intervention was delivered and over what period, including the number of sessions, timing, duration, and intensity or dose	Once. 12 months.
9	If the intervention was planned to be personalized, tailored, or adapted, describe what was adapted, why, when, and how	Not
10	If the intervention was modified during the course of the study, describe the changes (what, why, when, and how)	Not
11	If adherence to or fidelity of the intervention was assessed, describe how	NA

	and by whom it was evaluated	
12	If adherence to or fidelity to the intervention was assessed, describe the extent to which it was delivered as planned.	NA

Key: R30

1	Name of intervention	We Chat
2	Describe the theoretical framework or the purpose of the essential components of the intervention	Intervention based on family support and risk perception can enhance type 2 diabetes mellitus (T2DM) patients' self-care activities. In addition, eHealth education is considered to improve family members' support for patients with T2DM. However, there is little evidence from rigorously designed studies on the effectiveness of an intervention combining these approaches.
3	Materials	The study established an official WeChat account called Jiading Sugar Steward, which included 3 modules: blood glucose data, complications, and notices.<br>The intervention was implemented by using this account to deliver intervention articles.
4	Procedures	This single-center, 2-parallel-group RCT was conducted between 2019 and 2020. Overall, 228 patients were recruited from Jiading District, Shanghai, and randomly divided into intervention and control groups. The intervention group received an eHealth family intervention based on community management via WeChat, whereas the control group received usual care. The primary outcome was the glycated hemoglobin (HbA1c) level of the patients with T2DM.
5	Describe the experience, training, and qualifications of the individual delivering the intervention	Only described as professional health providers.
6	Mode of intervention delivery	The study was a single-center, 2-parallel-group randomized controlled trial to assess the effectiveness of an eHealth family-based intervention. This structured intervention program assessed knowledge, attitude, and behaviors. The difference between the intervention and control groups was whether the family members of the patients with T2DM followed the official WeChat account.
7	Setting where the intervention is delivered	This randomized control trial was conducted in the central area of Jiading District, which includes 2 community health service centers: Jiading Town Community Health Service

		Center and Juyuan New District Community Health Service Center.
8	Describe the number of times the intervention was delivered and over what period, including the number of sessions, timing, duration, and intensity or dose	Once. 12 months.<br>This single-center, 2-parallel-group RCT was conducted between 2019 and 2020. Overall, 228 patients were recruited from Jiading District, Shanghai, and randomly divided into intervention and control groups. The intervention group received an eHealth family intervention based on community management via WeChat, whereas the control group received usual care. The primary outcome was the glycated hemoglobin (HbA1c) level of the patients with T2DM, and the secondary outcomes were self-management behavior (general and specific diet, exercise, blood sugar testing, foot care, and smoking), risk perception (risk knowledge, personal control, worry, optimism bias, and personal risk), and family support (supportive and nonsupportive behaviors).
9	If the intervention was planned to be personalized, tailored, or adapted, describe what was adapted, why, when, and how	Not
10	If the intervention was modified during the course of the study, describe the changes (what, why, when, and how)	Not
11	If adherence to or fidelity of the intervention was assessed, describe how and by whom it was evaluated	NA
12	If adherence to or fidelity to the intervention was assessed, describe the extent to which it was delivered as planned.	NA

Key: 33

1	Name of intervention	IoT
2	Describe the theoretical framework or the purpose of the essential components of the intervention	Behavioral changes are essential to achieve efficient glycemic control in people with diabetes. Diabetes self-management education and support are the most important to promote behavioral changes in exercise and diet, leading to a high adherence to medical nutrition therapy, increased physical

		activity, and finally reduced glycated hemoglobin (HbA1c) levels.<br>Furthermore, multifaceted behavioral interventions can significantly reduce the non-attendance for regular visits and improve the quality of diabetes care in type 2 diabetes patients.<br>However, evidence-based lifestyle interventions are expensive and require the extensive use of human resource.
3	Materials	The PRISM-J study was an open-label, randomized, parallel-group trial of IoT-based approach vs conventional therapy for type 2 diabetes. The trial design and protocol of this study and IoT-based approaches for lifestyle self-monitoring, feedback message generation, and data collection have been reported previously.
4	Procedures	According to the recommendations for self-monitoring of personal health record in diabetes18–20, all the patients were provided with devices with IoT functions, including a weight and body composition monitor, blood pressure monitor, and activity monitor, and were free to use these devices as reported previously. Patients in the ITG were instructed to use the devices and could check the obtained PHR on their smartphone through Omron connect. The PHR were regularly sent to the device database (Omron cloud), connected to Shichifukujin cloud; feedback messages that can promote behavioral changes in accordance with the Japanese guidelines were<br>automatically generated by the Shichifukujin application (SFJA)nand sent to the patient twice a week (Figure 1). In contrast, as the percentage of smartphone users in Japan was already over 60% as of 201724, patients in the CTG were not restricted in using smartphones and as Omron Connect was a commercial application, they were consequently able to check their PHR on their smartphone, although they were not educated on how to<br>use these devices and the application. Moreover, they were not allowed to receive feedback messages from the SFJA. Adjustments of diabetes medications were left to the discretion of the attending physician.
5	Describe the experience, training, and qualifications of the individual delivering the intervention	Only described as doctors.
6	Mode of intervention delivery	Patients were randomly assigned in a 1:1 ratio to either the IoT intervention group (ITG) or the conventional therapy group (CTG) using stratified blocked randomization (block size set to10), based on age (≥50 or <50 years), sex (male/female), body mass index (BMI; ≥25 or <25 kg/m2), and hemoglobin

		A1c (HbA1c; ≥8.0 [64 mmol/mol], <8.0%), that was generated by the statistician of the study (HO). Neither patients nor investigators were masked to treatment group assignment. The study included a 52-week intervention period and a maximum 52 week observational period.
7	Setting where the intervention is delivered	Participants were recruited from 93 hospitals/ clinics and 113 health insurance societies in Japan
8	Describe the number of times the intervention was delivered and over what period, including the number of sessions, timing, duration, and intensity or dose	Once. 52 weeks.<br>From January 2018 to December 2018, 1,159 patients were enrolled and randomized while the target sample size was 2,000.<br>Patients who had their glucose values and other clinical indicators checked received information and feedback twice a week.
9	If the intervention was planned to be personalized, tailored, or adapted, describe what was adapted, why, when, and how	Not
10	If the intervention was modified during the course of the study, describe the changes (what, why, when, and how)	Not
11	If adherence to or fidelity of the intervention was assessed, describe how and by whom it was evaluated	NA
12	If adherence to or fidelity to the intervention was assessed, describe the extent to which it was delivered as planned.	NA

References of articles reviewed using the Jadad scale

R8
Amante DJ, Harlan DM, Lemon SC, McManus DD, Olaitan OO, Pagoto SL, et al. Evaluation of a diabetes remote monitoring program facilitated by connected glucose meters for patients with poorly controlled type 2 diabetes: randomized crossover trial. JMIR Diabetes. 2021;6(1):e25574. doi:10.2196/25574.

R12
Gong E, Baptista S, Russell A, Scuffham P, Riddell M, Speight J, et al. My Diabetes Coach, a mobile app-based interactive conversational agent to support type 2 diabetes self-management: randomized effectiveness–implementation trial. J Med Internet Res. 2020;22(11):e20322. doi:10.2196/20322.

R15
Kim Y, Lee H, Seo JM. Integrated diabetes self-management program using smartphone application: a randomized controlled trial. West J Nurs Res. 2022;44(4):383–94. doi:10.1177/0193945921994912.

R18
Xia SF, Maitiniyazi G, Chen Y, Wu XY, Zhang Y, Zhang XY, et al. Web-based TangPlan and WeChat combination to support self-management for patients with type 2 diabetes: randomized controlled trial. JMIR Mhealth Uhealth. 2022;10(3):e30571. doi:10.2196/30571.

R21
Lin K, Zhang W, He F, Shen J. Evaluation of the clinical efficacy of the treatment of overweight and obesity in type 2 diabetes mellitus by a telemedicine management system based on Internet of Things technology. Comput Intell Neurosci. 2022;2022:8149515. doi:10.1155/2022/8149515.

R22
Lu Z, Li Y, He Y, Zhai Y, Wu J, Wang J, et al. Internet-based medication management services improve glycated hemoglobin levels in patients with type 2 diabetes. Telemed J E Health. 2021;27(6):686–93. doi:10.1089/tmj.2020.0123.

R23
Ramallo-Fariña Y, García-Bello MA, García-Pérez L, Boronat M, Wägner AM, Rodríguez-Rodríguez L, et al. Effectiveness of internet-based multicomponent interventions for patients and health care professionals to improve clinical outcomes in type 2 diabetes evaluated through the INDICA study: a multiarm cluster randomized controlled trial. JMIR Mhealth Uhealth. 2020;8(11):e18922. doi:10.2196/18922.

R24
Woodard L, Amspoker AB, Hundt NE, Gordon HS, Hertz B, Odom E, et al. Comparison of collaborative goal setting with enhanced education for managing diabetes-associated distress and hemoglobin A1c levels: a randomized clinical trial. JAMA Netw Open. 2022;5(5):e229975. doi:10.1001/jamanetworkopen.2022.9975.

R27
Han CY, Zhang J, Ye XM, Lu JP, Jin HY, Xu WW, et al. Telemedicine-assisted structured self-monitoring of blood glucose in management of type 2 diabetes mellitus: results of a randomized clinical trial. BMC Med Inform Decis Mak. 2023;23(1):182. doi:10.1186/s12911-023-02283-4.

R28
Guo M, Meng F, Guo Q, Bai T, Hong Y, Song F, et al. Effectiveness of mHealth management with an implantable glucose sensor and a mobile application among Chinese adults with type 2 diabetes. J Telemed Telecare. 2023;29(8):632–40. doi:10.1177/1357633X211020261.

R29
Jia W, Zhang P, Zhu D, Duolikun N, Li H, Bao Y, et al. Evaluation of an mHealth-enabled hierarchical diabetes management intervention in primary care in China (ROADMAP): a cluster randomized trial. PLoS Med. 2021;18(9):e1003754. doi:10.1371/journal.pmed.1003754.

R30
Feng Y, Zhao Y, Mao L, Gu M, Yuan H, Lu J, et al. Effectiveness of an eHealth family-based intervention program in patients with uncontrolled type 2 diabetes mellitus in the community via WeChat: a randomized controlled trial. JMIR Mhealth Uhealth. 2023;11:e40420. doi:10.2196/40420.

R33
Bouchi R, Izumi K, Ishizuka N, Uemura Y, Ohtsu H, Miyo K, et al. Internet of Things–based approach for glycemic control in people with type 2 diabetes: a randomized controlled trial. J Diabetes Investig. 2024;15(9):1287–96. doi:10.1111/jdi.14227.

S4.- JADAD SCORES FOR SELECTED ARTICLES

Question<br>**Yes: 1 point**<br>**No: 0 points**	R1	R2	R3	R4	R5	R6	R7	R8	R9	R 10	R 11	R 12	R 13	R 14	R 15	R 16	R 17	R 18	R 19	R 20	R 21	R 22	R 23	R 24	R 25	R 26	R 27	R 28	R 29	R 30	R 31	R 32	R 33
**Is the study described as randomized?**	0	1	0	1	0	0	1	1	1	1	1	1	0	1	1	1	1	1	0	1	1	1	1	1	1	1	1	1	1	1	1	1	1
**Is the method used to generate the randomization sequence described and is this method appropriate?"**	0	0	0	0	0	0	1	1	0	1	0	1	0	0	1	0	0	1	1	1	1	1	1	1	1	1	1	1	1	1	1	1	1
**Is the method used to generate the randomization sequence appropriate?**	-1	-1	-1	-1	-1	-1	1	1	-1	1	-1	1	-1	-1	1	1	1	1	-1	-1	1	1	1	1	1	1	1	1	1	1	1	1	1
**Is the study described as double-blind?**	0	0	0	0	0	0	0	0	0	0	0	0	0	0	1	0	0	0	0	0	0	0	0	0	0	0	0	0	0	0	0	0	0
**Is the method of blinding (or masking) described and is this method appropriate?**	0	0	0	0	0	0	0	0	0	0	0	0	0	0	1	0	0	0	0	0	0	1	0	1	0	0	1	0	1	0	0	0	1
**"Is the blinding (masking) method appropriate?"**	-1	-1	-1	0	-1	-1	-1	-1	-1	-1	-1	-1	-1	-1	1	-1	-1	-1	0	0	-1	1	-1	1	-1	-1	1	-1	1	-1	-1	-1	-1
**"Is there a description of losses to follow-up and withdrawals?"**	1	0	0	1	0	0	0	1	0	0	0	1	1	1	0	1	1	1	0	0	1	0	1	1	0	0	1	1	1	1	0	0	1

Note: Selected articles with less risk of bias are in red.
Jadad Scale for RCT Validation: assigns negative points when the method of randomization is not described or is deemed inappropriate for the study design. Likewise, it penalizes the absence of blinding in the trial's design and implementation. In this way, the scale applies more stringent criteria for assessing the methodological quality of the selected trials.

References of articles reviewed using the Jadad scale

R1
Summers C, Tobin S, Unwin D. Evaluation of the Low Carb Program digital intervention for the self-management of type 2 diabetes and prediabetes in an NHS England general practice: Single-arm prospective study. JMIR Diabetes [Internet]. 2021;6(3):e25751. Available at: http://dx.doi.org/10.2196/25751
R2
Jonusas J, Aleknavicius K, Valinskas S. Klinio mobile app for diabetes self-care: A pilot study of HbA1c improvement in type 2 diabetes patients. Smart Health [Internet]. 2023;29(100404):100404. Available at: http://dx.doi.org/10.1016/j.smhl.2023.100404
R3
Hsia J, Guthrie NL, Lupinacci P, Gubbi A, Denham D, Berman MA, et al. Randomized, controlled trial of a digital behavioral therapeutic application to improve glycemic control in adults with type 2 diabetes. Diabetes Care [Internet]. 2022;45(12):2976–81. Available at: http://dx.doi.org/10.2337/dc22-1099
R4
Zhong J, Zhang H, Li Z, Qian D, Zhang Y, Li C, et al. Effect of social app-assisted education and support on glucose control in patients with coronary heart disease and diabetes mellitus. Front Cardiovasc Med [Internet]. 2022;9:947130. Available at: http://dx.doi.org/10.3389/fcvm.2022.947130
R5
Heald AH, Roberts S, Albeda Gimeno L, Gilingham E, James M, White A, et al. A randomised control trial to explore the impact and efficacy of the healum collaborative care planning software and app on condition management in the Type 2 Diabetes mellitus population in NHS primary care. Diabetes Ther [Internet]. 2023;14(6):977–88. Available at: http://dx.doi.org/10.1007/s13300-023-01404-6
R6
Milani R, Chava P, Wilt J, Entwisle J, Karam S, Burton J, et al. Improving management of type 2 diabetes using home-based telemonitoring: Cohort study. JMIR Diabetes [Internet]. 2021;6(2):e24687. Available at: http://dx.doi.org/10.2196/24687
R7
Kusnanto, Widyanata KAJ, Suprajitno, Arifin H. DM-calendar app as a diabetes self-management education on adult type 2 diabetes mellitus: a randomized controlled trial. J Diabetes Metab Disord [Internet]. 2019;18(2):557–63. Available at: http://dx.doi.org/10.1007/s40200-019-00468-1
R8
Amante DJ, Harlan DM, Lemon SC, McManus DD, Olaitan OO, Pagoto SL, et al. Evaluation of a diabetes remote monitoring program facilitated by connected glucose meters for patients with poorly controlled type 2 diabetes: Randomized crossover trial. JMIR Diabetes [Internet]. 2021;6(1):e25574. Available at: http://dx.doi.org/10.2196/25574

R9
Mizokami-Stout K, Choi H, Richardson CR, Piatt G, Heisler M. Diabetes distress and glycemic control in type 2 diabetes: Mediator and moderator analysis of a peer support intervention. JMIR Diabetes [Internet]. 2021;6(1):e21400. Available at: http://dx.doi.org/10.2196/21400
R10
Zamanillo-Campos R, Fiol-deRoque MA, Serrano-Ripoll MJ, Mira-Martínez S, Ricci-Cabello I. Development and evaluation of DiabeText, a personalized mHealth intervention to support medication adherence and lifestyle change behaviour in patients with type 2 diabetes in Spain: A mixed-methods phase II pragmatic randomized controlled clinical trial. Int J Med Inform [Internet]. 2023;176(105103):105103. Available at: http://dx.doi.org/10.1016/j.ijmedinf.2023.105103
R11
Polanco MA. 64-LB: Long-term effectiveness of the my Dose Coach application on the maintenance of glycemic control in patients with type 2 Diabetes mellitus in uncontrolled. Diabetes [Internet]. 2022;71(Supplement_1). Available at: http://dx.doi.org/10.2337/db22-64-lb
R12
Gong E, Baptista S, Russell A, Scuffham P, Riddell M, Speight J, et al. My Diabetes Coach, a mobile app-based interactive conversational agent to support type 2 diabetes self-management: Randomized effectiveness-implementation trial. J Med Internet Res [Internet]. 2020;22(11):e20322. Available at: http://dx.doi.org/10.2196/203222.
R13
Zimmermann G, Venkatesan A, Rawlings K, Scahill MD. Improved glycemic control with a digital health intervention in adults with type 2 diabetes: Retrospective study. JMIR Diabetes [Internet]. 2021;6(2):e28033. Available at: http://dx.doi.org/10.2196/28033
R14
Chawla R, Jaggi S, Gupta A, Bantwal G, Patil S. Clinical utility of a digital therapeutic intervention in Indian patients with type 2 diabetes mellitus: 12-week prospective single-arm intervention study. JMIR Diabetes [Internet]. 2022;7(4):e41401. Available at: http://dx.doi.org/10.2196/41401
R15
Kim Y, Lee H, Seo JM. Integrated diabetes self-management program using smartphone application: A randomized controlled trial. West J Nurs Res [Internet]. 2022;44(4):383–94. Available at: http://dx.doi.org/10.1177/0193945921994912
R16
Whitehouse CR, Knowles M, Long JA, Mitra N, Volpp KG, Xu C, et al. Digital health and community health worker support for diabetes management: A randomized controlled trial. J Gen Intern Med [Internet]. 2023;38(1):131–7. Available at: http://dx.doi.org/10.1007/s11606-022-07639-6
R17
Finkelstein EA, Gardner DS-L, Tham KW, Gandhi M, Cheung YB, Bairavi J, et al. Effectiveness and cost-effectiveness of an app and rewards-based intervention in type 2 diabetes: A randomised

controlled trial. Diabetes Obes Metab [Internet]. 2025;27(2):729–39. Available at: http://dx.doi.org/10.1111/dom.16067

R18

Xia S-F, Maitiniyazi G, Chen Y, Wu X-Y, Zhang Y, Zhang X-Y, et al. Web-based TangPlan and WeChat combination to support self-management for patients with type 2 diabetes: Randomized controlled trial. JMIR MHealth UHealth [Internet]. 2022;10(3):e30571. Available at: http://dx.doi.org/10.2196/30571

R19

Kim G, Kim S, Lee Y-B, Jin S-M, Hur KY, Kim JH. A randomized controlled trial of an app-based intervention on physical activity and glycemic control in people with type 2 diabetes. BMC Med [Internet]. 2024;22(1):185. Available at: http://dx.doi.org/10.1186/s12916-024-03408-w

R20

Philis-Tsimikas A, Fortmann AL, Godino JG, Schultz J, Roesch SC, Gilmer TP, et al. Dulce Digital-Me: protocol for a randomized controlled trial of an adaptive mHealth intervention for underserved Hispanics with diabetes. Trials [Internet]. 2022;23(1):80. Available at: http://dx.doi.org/10.1186/s13063-021-05899-x

R21

Lin K, Zhang W, He F, Shen J. Evaluation of the clinical efficacy of the treatment of overweight and obesity in type 2 diabetes mellitus by the telemedicine management system based on the Internet of Things technology. Comput Intell Neurosci [Internet]. 2022;2022:8149515. Available at: http://dx.doi.org/10.1155/2022/8149515

R22

Lu Z, Li Y, He Y, Zhai Y, Wu J, Wang J, et al. Internet-based medication management services improve glycated hemoglobin levels in patients with type 2 diabetes. Telemed J E Health [Internet]. 2021;27(6):686–93. Available at: http://dx.doi.org/10.1089/tmj.2020.0123

R23

Ramallo-Fariña Y, García-Bello MA, García-Pérez L, Boronat M, Wägner AM, Rodríguez-Rodríguez L, et al. Effectiveness of internet-based multicomponent interventions for patients and health care professionals to improve clinical outcomes in type 2 diabetes evaluated through the INDICA study: Multiarm cluster randomized controlled trial. JMIR MHealth UHealth [Internet]. 2020;8(11):e18922. Available at: http://dx.doi.org/10.2196/18922

R24

Woodard L, Amspoker AB, Hundt NE, Gordon HS, Hertz B, Odom E, et al. Comparison of collaborative goal setting with enhanced education for managing diabetes-associated distress and hemoglobin A1c levels: A randomized clinical trial: A randomized clinical trial. JAMA Netw Open [Internet]. 2022;5(5):e229975. Available at: http://dx.doi.org/10.1001/jamanetworkopen.2022.9975

R25

Yang L, Xu J, Kang C, Bai Q, Wang X, Du S, et al. Effects of mobile phone-based telemedicine management in patients with type 2 diabetes mellitus: A randomized clinical trial. Am J Med Sci [Internet]. 2022;363(3):224–31. Available at: http://dx.doi.org/10.1016/j.amjms.2021.09.001
R26
Vaughan EM, Naik AD, Amspoker AB, Johnston CA, Landrum JD, Balasubramanyam A, et al. Mentored implementation to initiate a diabetes program in an underserved community: a pilot study. BMJ Open Diabetes Res Care [Internet]. 2021;9(1):e002320. Available at: http://dx.doi.org/10.1136/bmjdrc-2021-002320
R27
Han C-Y, Zhang J, Ye X-M, Lu J-P, Jin H-Y, Xu W-W, et al. Telemedicine-assisted structured self-monitoring of blood glucose in management of T2DM results of a randomized clinical trial. BMC Med Inform Decis Mak [Internet]. 2023;23(1):182. Available at: http://dx.doi.org/10.1186/s12911-023-02283-4
R28
Guo M, Meng F, Guo Q, Bai T, Hong Y, Song F, et al. Effectiveness of mHealth management with an implantable glucose sensor and a mobile application among Chinese adults with type 2 diabetes. J Telemed Telecare [Internet]. 2023;29(8):632–40. Available at: http://dx.doi.org/10.1177/1357633X211020261
R29
Jia W, Zhang P, Zhu D, Duolikun N, Li H, Bao Y, et al. Evaluation of an mHealth-enabled hierarchical diabetes management intervention in primary care in China (ROADMAP): A cluster randomized trial. PLoS Med [Internet]. 2021;18(9):e1003754. Available at: http://dx.doi.org/10.1371/journal.pmed.1003754
R30
Feng Y, Zhao Y, Mao L, Gu M, Yuan H, Lu J, et al. The effectiveness of an eHealth family-based intervention program in patients with uncontrolled type 2 diabetes mellitus (T2DM) in the community via WeChat: Randomized controlled trial. JMIR MHealth UHealth [Internet]. 2023;11:e40420. Available at: http://dx.doi.org/10.2196/40420
R31
Orozco-Beltrán D, Morales C, Artola-Menéndez S, Brotons C, Carrascosa S, González C, et al. Effects of a digital patient empowerment and communication tool on metabolic control in people with type 2 diabetes: The DeMpower multicenter ambispective study. JMIR Diabetes [Internet]. 2022;7(4):e40377. Available at: http://dx.doi.org/10.2196/40377
R32
White A, Bradley D, Buschur E, Harris C, LaFleur J, Pennell M, et al. Effectiveness of a diabetes-focused electronic discharge order set and postdischarge nursing support among poorly controlled hospitalized patients: Randomized controlled trial. JMIR Diabetes [Internet]. 2022;7(3):e33401. Available at: http://dx.doi.org/10.2196/33401

R33
Bouchi R, Izumi K, Ishizuka N, Uemura Y, Ohtsu H, Miyo K, et al. Internet of things-based approach for glycemic control in people with type 2 diabetes: A randomized controlled trial. J Diabetes Investig [Internet]. 2024;15(9):1287–96. Available at: http://dx.doi.org/10.1111/jdi.14227

S5.- GRADE SUMMARY

According to the GRADE assessment, the certainty of evidence for HbA1c reduction associated with digital health interventions compared with usual care was rated as moderate.

Outcome	Number of studies	Number of participants	Effect estimates	Certainty of evidence
Reduction in HbA1c (%)	13 RCT	approximately 20,000	Mean difference −1.08% (95% CI −1.18 to −0.99)	Moderate

Indicates that the true effect is likely to be close to the estimate of the effect, but there is a possibility that it is substantially different.

GRADE Justification

The certainty of evidence was downgraded by one level due to serious inconsistency, as indicated by high unexplained heterogeneity across studies.

The certainty was further downgraded by one level due to some concerns regarding risk of bias, mainly related to limited blinding and incomplete methodological reporting in several trials.

No downgrading was applied for indirectness, as the population, intervention, comparator, and outcome was directly relevant to the research question.

No downgrading was applied for imprecision, given the large sample size and narrow confidence intervals.

No downgrading was applied for publication bias, as funnel plot inspection and trim-and-fill analysis did not suggest missing studies.
